# Epigenetic profiles signify cell fate plasticity in unipotent spermatogonial stem and progenitor cells

**DOI:** 10.1038/ncomms11275

**Published:** 2016-04-27

**Authors:** Ying Liu, Eugenia G. Giannopoulou, Duancheng Wen, Ilaria Falciatori, Olivier Elemento, C. David Allis, Shahin Rafii, Marco Seandel

**Affiliations:** 1 Department of Medicine, Division of Regenerative Medicine, Ansary Stem Cell Institute, Weill Cornell Medical College, 1300 York Avenue, New York, New York 10065, USA; 2 Chromatin Biology and Epigenetics, The Rockefeller University, New York, New York 10065, USA; 3 Biological Sciences Department, New York City College of Technology, City University of New York, Brooklyn, New York 11201, USA; 4 Arthritis and Tissue Degeneration Program and the David Z. Rosensweig Genomics Research Center, Hospital for Special Surgery, New York, New York 10021, USA; 5 Ronald O. Perelman and Claudia Cohen Center for Reproductive Medicine, Weill Cornell Medical College, New York, New York 10065, USA; 6 Cancer Research UK Cambridge Institute, Li Ka Shing Centre, University of Cambridge, Cambridge CB2 0RE, UK; 7 HRH Prince Alwaleed Bin Talal Bin Abdulaziz Alsaud Institute for Computational Biomedicine, Weill Cornell Medical College, New York, New York 10065, USA; 8 Department of Physiology and Biophysics, Weill Cornell Medical College, New York, New York 10065, USA; 9 Department of Surgery, Weill Cornell Medical College, New York, New York 10065, USA

## Abstract

Spermatogonial stem and progenitor cells (SSCs) generate adult male gametes. During *in vitro* expansion, these unipotent murine cells spontaneously convert to multipotent adult spermatogonial-derived stem cells (MASCs). Here we investigate this conversion process through integrative transcriptomic and epigenomic analyses. We find in SSCs that promoters essential to maintenance and differentiation of embryonic stem cells (ESCs) are enriched with histone H3-lysine4 and -lysine 27 trimethylations. These bivalent modifications are maintained at most somatic promoters after conversion, bestowing MASCs an ESC-like promoter chromatin. At enhancers, the core pluripotency circuitry is activated partially in SSCs and completely in MASCs, concomitant with loss of germ cell-specific gene expression and initiation of embryonic-like programs. Furthermore, SSCs *in vitro* maintain the epigenomic characteristics of germ cells *in vivo*. Our observations suggest that SSCs encode innate plasticity through the epigenome and that both conversion of promoter chromatin states and activation of cell type-specific enhancers are prominent features of reprogramming.

## Abstract (web summary)

Spermatogonial stem cells (SSCs) spontaneously convert to multipotent adult spermatogonial-derived stem cells (MASCs). Here, the authors reveal the dynamics of bivalent histone H3-lysine4 and -lysine27 methylation signatures at somatic gene promoters in SSCs and ESC-like promoter chromatin states in MASCs.

## Graphical abstract

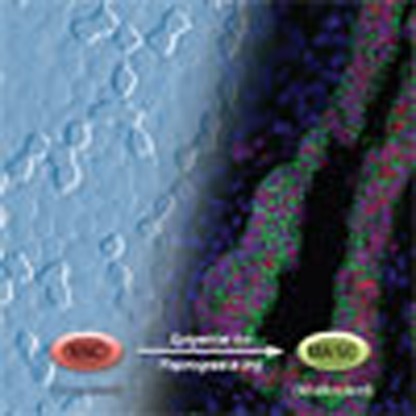

As the precursors of germ cells, mammalian spermatogonial stem and progenitor cells (SSCs) undergo unipotent differentiation in the adult male gonad, while still possessing the ultimate developmental potency to propagate across generations. During *in vitro* expansion, mouse SSCs, despite being unipotent, are uniquely capable of abrogating lineage commitment and spontaneously converting to multipotent adult spermatogonial-derived stem cells (MASCs), which share many features with pluripotent embryonic stem cells (ESCs) derived from the inner cell mass (ICM), including the capacity to induce teratomas and contribute to chimeric animals (Fig. 1a)^1^,^2^. To date, this is the only known spontaneous reprogramming event that converts unipotent adult stem cells back to a near-pluripotent state without delivery of exogenous genes or gene products, which distinguishes it from transcription factor-driven conversion of fibroblasts to induced pluripotent stem (iPS) cells^3^,^4^. These observations indicate that intrinsic genetic and epigenetic features are responsible for reprogramming of SSCs. However, SSC conversion into MASCs is a rare event, and the underlying mechanisms remain largely unknown.

One possible explanation for the spontaneous loss of lineage commitment is that SSCs may preserve a latent ESC-like gene expression programme. Indeed, upon germline specification in the mouse embryo, somatic genes are mainly repressed in primordial germ cells (PGCs), while several ESC signature transcription factors exhibit transcriptional activation and their expressions are preserved at modest levels in spermatogonia, which include SSCs in the adult testis^5^,^6^,^7^. For example, SSCs express *Pou5f1* (also known as *Oct4*), which forms the core pluripotency circuitry with *Sox2* and *Nanog* in ESCs to sustain stem cell self-renewal and control the expression of many differentiation genes^8^,^9^. As the precursors of all subsequent germ cells, SSCs also express spermatogenesis-specific genes (for example, *Zbtb16*, *Piwil2*, and *Piwil4*) but repress regulators of somatic cell development (for example, *Foxd3* and *Lhx2*)^10^,^11^,^12^.

Among other potential regulators that enable spontaneous conversion of SSCs into MASCs, the covalent modification of histones plays a key role in cell-type specification^13^,^14^,^15^,^16^. Extensive studies have shown that histone modifications are closely associated with transcription^17^,^18^. Notably, trimethylation on histone H3 lysine 4 (K4me3) at promoters associates with gene expression, while polycomb repressive complex 2 (PRC2)-mediated trimethylation on histone H3 lysine 27 (K27me3) associates with gene silencing^18^. In ESCs, K4me3 and K27me3 co-localize at promoters of many developmental genes that control stem cell differentiation to both somatic and germline lineages^19^,^20^. Therefore, the K4me3+K27me3 ‘bivalent' modification is suggested to place promoters in a poised state, while its resolution to a K4me3 or K27me3 univalent modification state is generally believed to direct gene activation or complete silencing, respectively, during stem cell differentiation^20^. Enhancers also confer *cis*-regulation to gene expression by recruiting specific transcription factors, and enhancer activity is tightly controlled in a cell-type-specific manner^21^. The presence of the acetylation on histone H3 lysine 27 (K27ac) at genomic regions is considered to denote active enhancers^21^,^22^.

On the basis of these observations, we hypothesize that the epigenetic landscape of SSCs is plastic, and under certain yet unrecognized conditions may cause conversion back to an ESC-like state. Here we perform transcriptome sequencing (RNA-seq) and find that MASCs are distinguished from SSCs by reactivation of somatic lineage-specific genes and repression of spermatogenesis regulators. To elucidate the epigenomic regulation underlying SSC conversion, we describe the results of chromatin immunoprecipitation followed by sequencing (ChIP-seq) on SSCs and MASCs to identify changes in transcription-associated histone modifications at both promoters and enhancers of reprogrammed genes. This genome-wide study reveals that long-term *in vitro*-cultured SSCs, which are capable of unipotent differentiation to all germ cells in mouse testicular transplantation, closely align with MASCs and ESCs in global gene expression and histone modification. In SSCs and differentiating germ cells, unlike differentiated somatic cells (for example, fibroblasts), K4me3 and K27me3 co-localize to a significant number of developmental gene promoters, and half of these promoters are bivalently modified in both MASCs and ESCs. Notably, in MASCs, which function like pluripotent stem cells (for example, in blastocyst chimerism), K27me3 is erased from many genes crucial to early embryogenesis and stem cell maintenance but is acquired at promoters of most germ cell-specific genes. These selective epigenetic alterations closely correlate with gene expression changes and endow MASCs with an ESC-like promoter chromatin. This phenomenon distinguishes SSC conversion from fibroblast to iPS cell reprogramming, which involves global epigenetic changes and reconstitution of promoter bivalency. At enhancer loci, we note that the germ cell epigenetic ‘signature' is largely lost in MASCs compared with SSCs, while ESC-specific enhancers are partially activated after reprogramming. Notably, unipotent SSCs share substantial enhancer activity with multipotent MASCs and pluripotent ESCs. These shared active enhancers are predicted targets of many ESC signature transcriptional regulators, including the core pluripotency regulators, such as *Pou5f1*, *Sox2* and *Nanog*. Taken together, our results reveal the topography of global histone modifications in mouse SSCs and MASCs, and indicate developmental lineage-associated epigenetic signature changes before and after spontaneous SSC cell fate transitions.

## Results

### MASCs share their transcriptome and epigenome with ESCs

**Figure 1:**
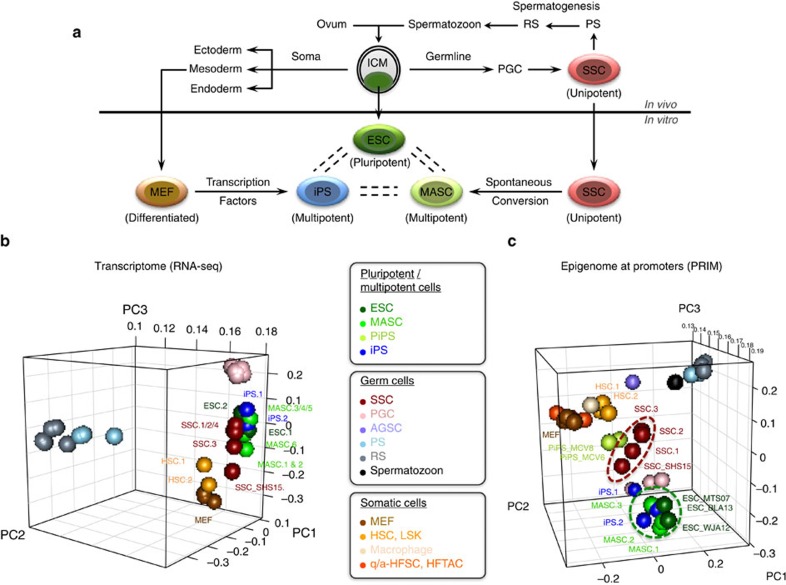
Comparison of transcriptomes and epigenomes among different cell types. (**a**) Cell type and developmental potency. Dark green, ESC and inner cell mass (ICM); green, MASC; red, SSC; blue, iPS cell; brown, MEF. Other male germ cells include PGC, pachytene spermatocyte (PS), round spermatid (RS) and spermatozoon. (**b**) Three-dimensional (3D) PCA plot based on mRNA expression of all protein-coding and noncoding genes. Dark green, ESCs; green, MASCs; blue, iPS; light green, incompletely reprogrammed MEFs (PiPS); red, SSC; pink, PGCs; brown, MEFs; dark orange, quiescent/activated-hair follicle stem cell (q/a-HFSC) and hair follicle transient-amplifying matrix cell (HFTAC); orange, HSC from culture or fluorescent-activated cell sorting (FACS)-isolated lineage^−^, Sca-1^+^ and c-kit^+^ (LSK) cells; light orange, macrophage; slate blue, FACS-isolated Thy1+ adult germline stem cell (AGSC); sky blue, PS; grey, RS; black, spermatozoon. (**c**) 3D PCA plot based on PRIMs of all protein-coding and noncoding gene promoters with K4me3 and/or K27me3 modification. PRIM is calculated by read intensity ratio between K4me3 and K27me3 peaks at the same promoter region (log2(K4me3/K27me3)). Different cell types are distinguished by colours as in **b**.

We first compared global gene expression among MASCs, ESCs and iPS cells using hierarchical clustering of RNA-seq results collected from each cell type (Supplementary Data 1). To ensure consistent transcriptional profiling, six MASC lines and two ESC lines of different genetic backgrounds were subcloned and analysed. The iPS cell lines were generated by doxycycline induction in E13.5 mouse embryonic fibroblasts (MEFs) engineered with a polycistronic OSKM 4F2A cassette (*tetO-4F2A*)^23^. Both MASC and iPS cell lines could efficiently form tri-lineage teratomas, a key criterion for pluripotency. Consistent with previous studies^1^,^2^, MASCs were transcriptionally similar to ESCs and iPS cells, but distinct from differentiating germ cells pachytene spermatocytes^24^,^25^ and round spermatids^24^,^26^,^27^, as well as from cells committed to somatic lineages, like MEFs^28^ and haematopoietic stem and progenitor cells (HSCs) (Fig. 1b and Supplementary Fig. 1A).

To study the epigenetic characteristics of different cell types, we performed ChIP-seq for selected histone modifications that are closely associated with transcriptional activation (K4me3 and K27ac) and repression (K27me3). ChIP-seq results from MASCs, ESCs^29^,^30^,^31^, completely reprogrammed MEFs (iPS_MCV81, iPS.1 and iPS.2)^32^, incompletely reprogrammed MEFs (PiPS_MCV6 and PiPS_MCV8)^32^ and differentiated somatic^29^,^32^,^33^,^34^ or germ cells^24^,^26^,^27^,^35^ were collected from our lab or public databases (Supplementary Data 2). In each cell line, promoters were interrogated for significant K4me3 or K27me3 modification peaks with ChIPseeqer-2.0 (false discovery rate (FDR)<0.05)^36^. We then generated a statistical vector that evaluates the epigenetic likelihood of transcription at each promoter, represented by the peak intensity ratio between the two modifications K4me3 and K27me3 at the same promoter (see Methods). This vector was referred to as the promoter ChIP-seq read intensity ratio for histone modification (PRIM). Principal component analysis (PCA) analysis with PRIM values showed that MASCs, like iPS cells, closely resembled ESCs in terms of global promoter K4me3 and K27me3 modifications (Fig. 1c). This epigenomic similarity was also identified in PGCs isolated from E12.5–E14.5 embryos (Fig. 1c (pink)), which have the capability to achieve pluripotency during *in vitro* expansion^37^,^38^. For comparison, incompletely reprogrammed MEFs (PiPS_MCV6 and PiPS_MCV8) were epigenomically closer to MEFs than to iPS cells, MASCs and ESCs (Fig. 1c and Supplementary Fig. 1B (light green)). Similar results were observed when we repeated the analyses with only our in-house cell lines (Supplementary Figs 1 and 2). The robustness of transcriptomes and epigenomes of individual cell types was confirmed by the Pearson's correlation coefficients (*r*) between in-house and published data (Supplementary Fig. 3). Thus, our results demonstrate that MASCs, like fully reprogrammed MEF-derived iPS cells, closely resemble ESCs with respect to not only global gene expression but also promoter histone modification and relative K4me3/K27me3 enrichment.

### SSCs are epigenomically similar to multipotent cell types

In comparison with differentiated somatic or germ cells in our study, we noticed that the SSC transcriptome was very close to MASCs, ESCs and iPS cells, irrespective of genetic background or source of data (Fig. 1b and Supplementary Fig. 1A (red)). Intriguingly, similar associations were also identified in our study of the epigenome. PCA analysis with PRIM from all promoters revealed that *in vitro*-cultured SSCs were epigenomically distant from any adult germ cells, including pachytene spermatocytes^24^,^27^, round spermatids^24^,^26^,^27^ and mature spermatozoa^26^, as well as from somatic cells like HSCs, macrophages, quiescent/activated-hair follicle stem cells and hair follicle transient-amplifying matrix cells^34^. Notably, the promoter epigenome of unipotent SSCs closely aligned with multipotent and pluripotent cells, including ESCs^29^,^30^,^31^, MASCs and iPS cells^32^ (Fig. 1c). Similar to SSCs, incompletely reprogrammed MEFs (PiPS_MCV6 and PiPS_MCV8) were positioned in the PCA analysis at an intermediate point between MEFs and iPS cells (Fig. 1c), consistent with their partial reprogramming status, as has been reported^32^.

### ESC-like bivalent promoter modifications in SSCs and MASCs

**Figure 2:**
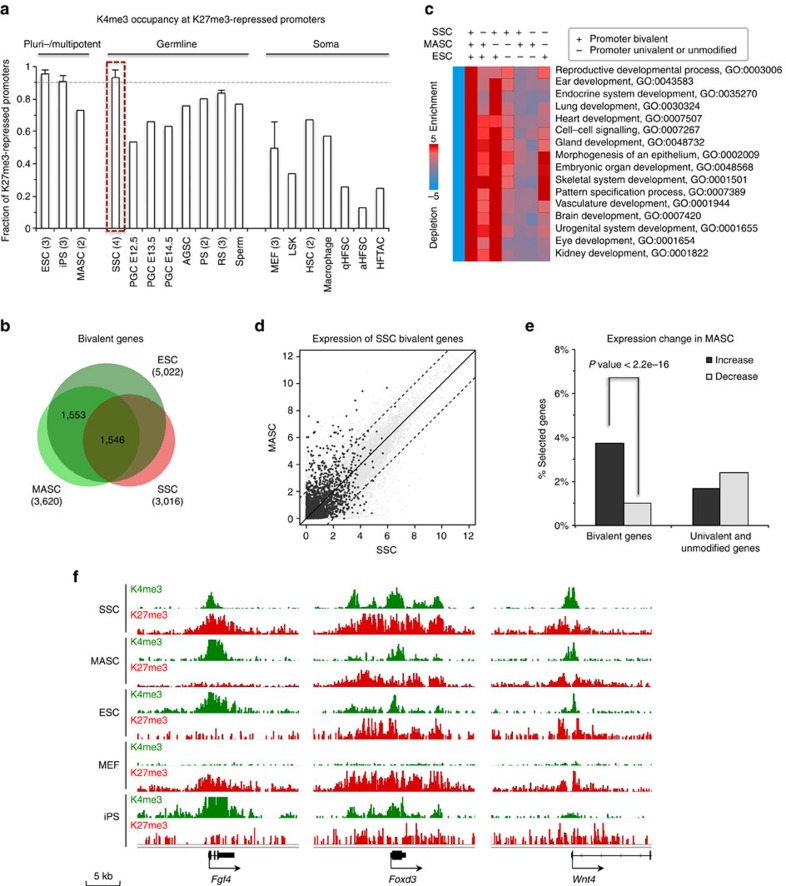
ESC-like bivalent promoter modifications are largely preserved in SSCs and selectively activated in MASCs. (**a**) Fraction of K4me3+K27me3 bivalent promoters within K27me3-marked promoters. For each cell type with at least three biological replicates (Supplementary Data 2) from different cell lines or resources, results are presented as mean values and s.d.'s. Number in brackets, sample size. Grey dashed line, fraction=0.9. (**b**) Overlap of bivalent genes identified by peak detection in SSCs, MASCs and ESCs (see Methods). (**c**) GO enrichment in bivalent genes shared in or unique to SSCs, MASCs and ESCs. ‘+' denotes promoter bivalency detected in each cell type. Enrichment and depletion of specific GO functions (shown on the right) were measured by hypergeometric *P* values (log10-transformed). The first column contains genes that do not belong to any of the measured classes and is used as a control gene list. Red, over-representation; blue, under-representation. (**d**) Comparison of global gene expression profiles between SSCs and MASCs. Black dots, SSC bivalent genes identified by peak detection; dashed line, cutoff of two-fold (log2) expression difference between SSCs and MASCs. (**e**) Percentage of genes with expression increase (black) or decrease (grey) in MASCs compared to SSCs for SSC bivalent genes (3,016 genes with both K4me3 and K27me3 modifications at promoters) and SSC univalent and unmodified genes (29,565 genes modified with either K4me3, K27me3 or neither of the two modifications at promoters). *P* value <2.2e-16 by Fisher's exact test (one-sided). (**f**) Promoter modification at selected genes. Green, K4me3; red, K27me3. K4me3 track range, 0–1; K27me3 track range, 0–0.5.

By using peak detection, we found that K4me3+K27me3 bivalent histone modifications were significantly enriched at many promoters in SSCs, the precursors of MASCs. In contrast, this ESC pluripotency-associated epigenetic signature was markedly less prevalent in the adult somatic stem and differentiated cell types we analysed (Supplementary Fig. 4A and Supplementary Data 3). Notably, bivalent promoters made up over 90% of the K27me3-marked promoters in SSCs, in stark contrast to other somatic cell types (Fig. 2a). Similarly, prominent coexistence of K4me3 and K27me3 histone modifications was detected only in multipotent and pluripotent cells (ESCs, MASCs and iPS cells) or in differentiating germ cells, but not in somatic cells (Fig. 2a). In somatic and germ cells, bivalent promoter modifications were mainly enriched at developmental genes that regulate their committed lineages, respectively. However, a broad range of embryonic developmental functions were identified in the bivalent genes in unipotent SSCs, as had been found in ESCs, MASCs and iPS cells (Supplementary Fig. 4B).

We noticed that over half (1,546/3,016) of SSC bivalent genes also possessed bivalent promoter modifications in MASCs and ESCs (Fig. 2b and Supplementary Data 4); such genes regulate embryonic development into both germline and somatic lineages (Fig. 2c). Although SSC bivalent genes mainly exhibited low expression in MASCs, a few were significantly activated in MASCs (Fig. 2d,e and Supplementary Data 4). Conversely, we only identified 396 promoters with bivalent modifications shared among ESCs, MEFs and iPS cells (Supplementary Fig. 5A and Supplementary Data 4). There were several pluripotency and early development regulators that carried both K4me3 and K27me3 modifications in SSCs but only possessed K27me3 modification in MEFs (for example, *Fgf4*, *Foxd3* and *Wnt4*; Fig. 2f). Unlike MASCs, iPS cells did not completely establish bivalency for differentiation, as had been suggested by the substantial enrichment of developmental functions in the 2,958 bivalent genes only identified in ESCs (Supplementary Fig. 5B). During cell reprogramming, MEF bivalent genes displayed less transcriptional bias than those in SSCs (Supplementary Fig. 5C,D and Supplementary Data 4). These results suggest both that unipotent SSCs possess a plastic chromatin configuration characterized by bivalent histone modifications, a feature conserved with pluripotent cell types, and that bivalent genes in SSCs are generally poised for activation before converting to MASCs. These epigenetic characteristics make SSC reprogramming distinct from iPS formation.

### Transcriptional alterations following SSC reprogramming

**Figure 3:**
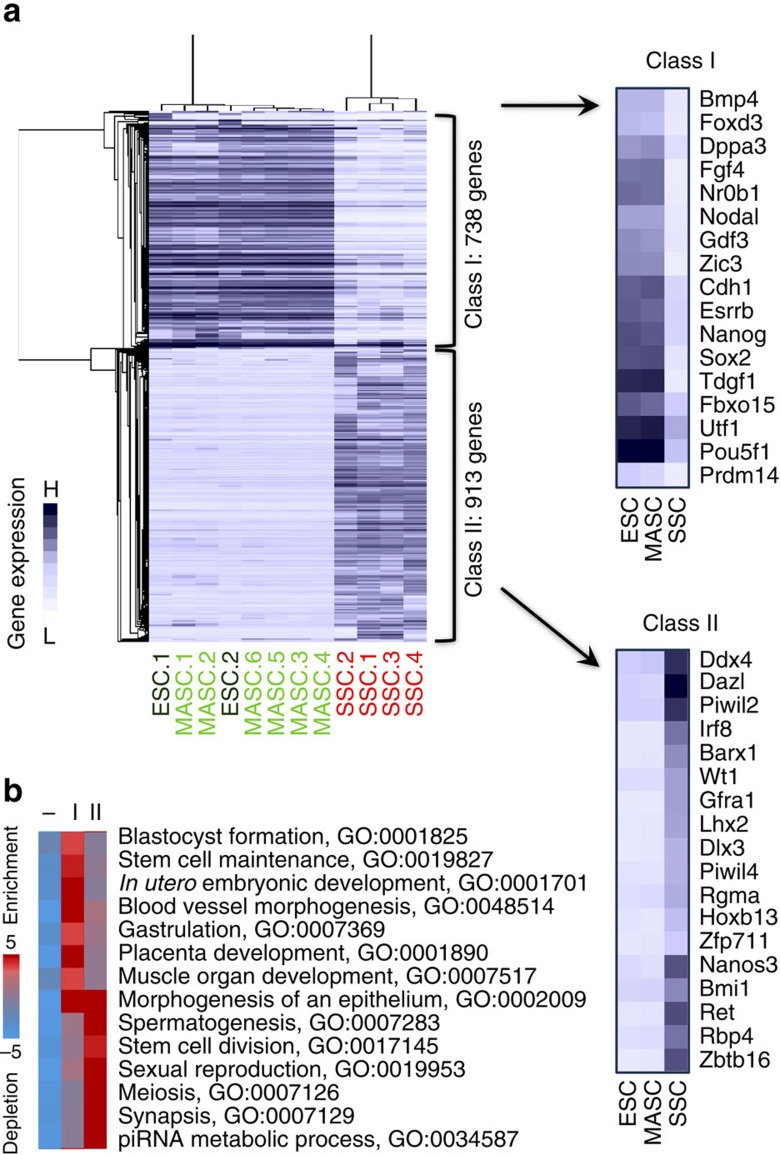
Activation of early embryonic genes and silencing of spermatogenesis-specific genes in MASCs. (**a**) Differential gene expression profiling among ESCs, MASCs and SSCs. Genes with over two-fold (log2) expression difference between any pair of samples were selected and subjected to hierarchical clustering. Right, average expression profiling in each cell type for example genes in each class. Blue and white indicate relative high and low gene expression, respectively. (**b**) GO enrichment in each gene class. Red and blue indicate relative over- and under-representation, respectively.

To elucidate the genomic regulation underlying spontaneous reprogramming of SSCs, we then focused on 1,651 genes differentially expressed (>2-fold change) among SSCs, MASCs and ESCs (Supplementary Data 5). Hierarchical clustering based on gene expression assigned them into two major classes: class I, 738 genes activated in MASCs and ESCs (and relatively repressed in SSCs); and class II, 913 genes repressed in MASCs and ESCs (Fig. 3a and Supplementary Data 5). Class I genes were mainly involved in ESC self-renewal and early embryonic development towards both somatic and germline lineages (for example, *Pou5f1*, *Nanog* and *Fgf4*; Fig. 3). Conversely, class II genes were highly expressed in SSCs and enriched with regulators of germ cell differentiation and meiosis (for example, *Zbtb16*, *Piwil2*, and *Piwil4*; Fig. 3). Thus, SSC reprogramming involved induction of early embryonic genes that function in ESC self-renewal and differentiation into both somatic and germline lineages, while expression of spermatogenesis-specific genes was reduced in MASCs.

### Bivalent promoter modification poises gene activation in SSCs

To understand how the transcriptomes and epigenomes coordinated for cell reprogramming, we then investigated K4me3 and K27me3 modifications at promoters of the differentially expressed genes among SSCs, MASCs and ESCs. To distinguish epigenetically repressive promoters (PRIM^low^, either K4me3+K27me3 or univalent K27me3) from active promoters (PRIM^high^, univalent K4me3), a specific PRIM cutoff was identified for each cell type (Supplementary Fig. 6).

**Figure 4:**
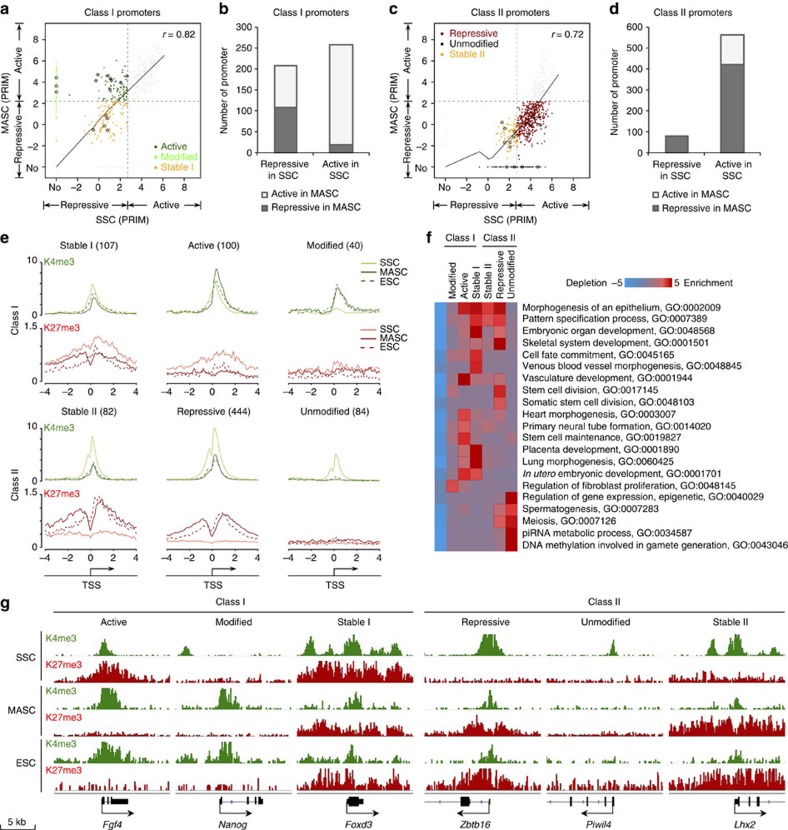
Promoter chromatin states of differentially expressed genes are selectively changed. (**a**) Comparison of chromatin states (PRIMs) between SSCs and MASCs for all class I gene promoters (grey dots). *x* axis, PRIMs from SSCs; *y* axis, PRIMs from MASCs. Black circles, representative class I genes from Fig. 3a; dashed black line, cutoff between active and repressive chromatin states; dark grey line, optimal curve fits to all class I gene promoters. *r*, correlation coefficient with all class I gene promoter PRIMs. On the basis of the cutoff value, three groups of promoters were selected: MASC^Active^ (dark green dots); MASC^Modified^ (light green dots); and MASC^Stable I^ (yellow dots). (**b**) Number of class I gene promoters changing between repressive and active chromatin states in SSCs and MASCs. Light grey, epigenetically active promoters in MASCs; dark grey, epigenetically repressive promoters in MASCs. (**c**) Comparison of chromatin states (PRIMs) between SSCs and MASCs for all class II gene promoters (grey dots). Black circles, representative class II from Fig. 3a; dark grey line, optimal curve fits to all class II gene promoters. *r*, correlation coefficient with all class II gene promoter PRIMs. On the basis of the cutoff value, three groups of promoters were selected: MASC^Repressive^ (dark red dots); MASC^Unmodified^ (black dots); and MASC^Stable II^ (yellow dots). (**d**) Number of class II gene promoters changing between repressive and active chromatin states in SSCs and MASCs. Light grey, epigenetically active promoters in MASCs; dark grey, epigenetically repressive promoters in MASCs. (**e**) Histone modification enrichment profiling for selected class I gene promoters (top) and class II gene promoters (bottom) (grouped as in **a**,**c**). Number in brackets, gene number in each group. Green, K4me3; red, K27me3; solid light line, SSCs; solid dark line, MASCs; dashed dark line, ESCs; arrow, direction of transcription; *x* axis, distance to TSS; *y* axis, average read density. TSS, transcription start site. (**f**) GO enrichment in selected genes. Genes are grouped by promoter PRIMs as in **a** and **c**. (**g**) Promoter modification at selected genes. Top, expression class and promoter type of each gene; green, K4me3; red, K27me3. K4me3 track range, 0–1; K27me3 track range, 0–0.5.

We hypothesized that reprogramming of SSCs without enforced expression of transcription factors would be associated with concomitant chromatin changes. Strikingly, genes that were transcriptionally activated in MASCs (class I) largely shared K4me3- and/or K27me3-marked promoter chromatin between SSCs and MASCs (Pearson's correlation with PRIM=0.82; Fig. 4a). Despite significantly increased expression after SSC reprogramming, we found that over half of the promoters that were epigenetically repressed in SSCs maintained low PRIM in MASCs (107 promoters, referred to as MASC^Stable I^; Fig. 4a (yellow dots), 4b and Supplementary Data 5). These MASC^Stable I^ genes included many of the bivalent genes found in SSCs by peak detection (Fig. 4e and Supplementary Data 5), and mainly function in stem cell differentiation and embryonic organ development into somatic lineages (for example, *Foxd3*; Fig. 4f,g).

We also found a small subset of class I gene promoters that either changed from a repressive state (SSCs) to an active state (MASCs), with complete erasure of K27me3 (100 promoters, MASC^Active^; Fig. 4a (dark green dots), 4e and Supplementary Data 5), or acquired *de novo* modifications, particularly K4me3, in MASCs (40 promoters, MASC^Modified^; Fig. 4a (light green dots), 4e and Supplementary Data 5). The MASC^Active^ and MASC^Modified^ subsets included many ESC signature genes associated with stem cell identity, such as *Fgf4* (MASC^Active^) and *Nanog* (MASC^Modified^; Fig. 4f,g). Compared with MASC^Stable I^ genes, MASC^Active^ and MASC^Modified^ genes were highly expressed in MASCs and ESCs, consistent with a strong impact of chromatin state changes on transcriptional regulation (Supplementary Fig. 7B). These three types of gene clusters included the majority of the pluripotency and developmental regulators activated in MASCs (Supplementary Fig. 7C).

Therefore, chromatin state changes were restricted to only ESC signature genes (class I, MASC^Active^ and MASC^Modified^), indicating that promoter chromatin state-associated transcriptional activation is both selective and gene specific. However, genes functioning in embryonic differentiation to somatic lineages maintained their bivalent promoter modifications in both SSCs and MASCs, consistent with recent observations in freshly isolated mouse spermatogonia^35^. Regulation of such genes during SSC reprogramming could be dominated by mechanisms that do not affect promoter histone modifications (for example, transcription factor binding at cell-type-specific enhancers).

### K27me3 marks germ cell-specific gene repression in MASCs

In contrast to class I, class II included 913 genes that were highly expressed in SSCs but downregulated in MASCs and ESCs (Fig. 3a). Correspondingly, most class II gene promoters shifted from an active towards a more repressive chromatin state after reprogramming (Fig. 4c). In particular, nearly half of the class II gene promoters were modified with concomitant *de novo* K27me3 and decreased K4me3 marks in MASCs (444 promoters, MASC^Repressive^; Fig. 4c (dark red dots), 4e and Supplementary Data 5), encompassing three-fourths of class II gene promoters that were epigenetically active in SSCs (Fig. 4d). Significant increases in K27me3 were also observed at promoters that exhibited both repressive chromatin states and relatively low expression in SSCs and MASCs (82 promoters, MASC^Stable II^; Fig. 4c (yellow dots), 4e, Supplementary Fig. 8B and Supplementary Data 5). These transcriptionally poised genes (MASC^Repressive^ and MASC^Stable II^) typically regulate embryonic differentiation towards both germline and somatic lineages (for example, *Zbtb16* and *Lhx2*; Fig. 4f,g). Furthermore, many spermatogenesis regulators exhibited substantial reductions in the K4me3 mark, yielding unmodified promoter chromatin in MASCs, such as *Piwil4* (MASC^Unmodified^; Fig. 4c (black dots), 4e–g and Supplementary Data 5). Expression of MASC^Unmodified^ and MASC^Stable II^ genes were generally much lower than the average class II genes in MASCs (Supplementary Fig. 8B).

Thus, the majority of somatic genes with bivalent promoter modifications in SSCs remained epigenetically repressed in MASCs, irrespective of their transcriptional activities (MASC^Stable I^ and MASC^Stable II^) after reprogramming. However, most ESC signature genes and germ cell-specific genes varied their K27me3 modifications at promoters, while a few of them either acquired or erased K4me3 modification in MASCs. These two histone modifications significantly affected the promoter chromatin states of only a small subset of transcriptionally activated genes (class I), but more prominently affected the repressed genes (class II).

### Reciprocal alterations in enhancer activity in MASCs

We extended our analysis of epigenetic regulation beyond promoters by using K27ac ChIP-seq, which marks active enhancers, defined as intergenic regions with K27ac modification and without K4me3 modification (K27ac+/K4me3−).

**Figure 5:**
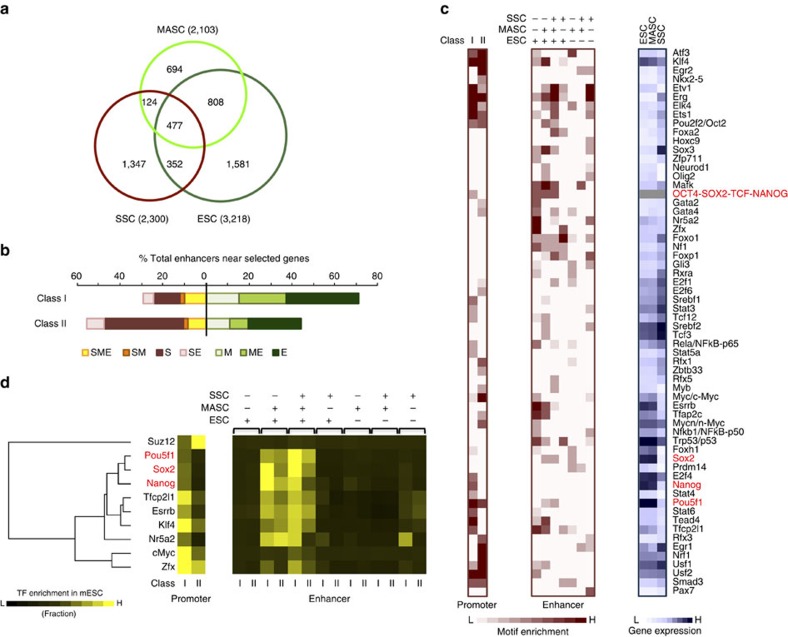
Transcriptional regulators potentially involved in chromatin state changes at promoters and enhancers. (**a**) Overlap of active enhancers in SSCs, MASCs, and ESCs. Number, number of enhancers in each subset. (**b**) Percentage of enhancers near Class I and II genes. S, enhancers active only in SSCs; SM, enhancers active in SSCs and MASCs; SE, enhancers active in SSCs and ESCs; SME, enhancers active in SSCs, MASCs, and ESCs; E, enhancers active only in ESCs; ME, enhancers active in MASCs and ESCs; M, enhancers active only in MASCs. (**c**) Enrichment of transcription factor binding motifs for promoters of expression class I and II (left) and enhancers active (+) or silent (−) in different cell types (middle). Each row represents a motif, and the expression of the corresponding transcriptional regulator is shown in the same row in the heatmap (right). Core pluripotency transcription factors are highlighted in red. Red and white in the heatmap indicate relative over- and under-representation of motif enrichment, respectively. Blue and white indicate relative high and low gene expression, respectively. (**d**) Enrichment of transcription factors at promoters and associated enhancers in ESCs. Promoters are grouped by expression class as in Fig. 3. Enhancers are grouped by activation (+) or silence (−) in different cell types (top) and expression class of associated promoters (bottom). Core pluripotency transcription factors are highlighted in red.

A total of 5,383 enhancers were found to be active in at least one of the three cell types (SSCs, MASCs and ESCs) and were associated with 1,651 differentially expressed genes (Supplementary Data 6). As predicted, MASCs, having lost lineage commitment, shared more active enhancers with pluripotent ESCs (808, ME enhancers) than with unipotent SSCs (124, SM enhancers; Fig. 5a). These ME enhancers, together with those only active in ESCs (E enhancers), dominated around somatic and ESC signature genes activated in MASCs (class I; Fig. 5b). Conversely, germ cell-specific genes that were repressed in MASCs (class II) mainly associated with enhancers uniquely active in SSCs (S enhancers; Fig. 5b).

Of note, over one-third of enhancers active in both MASCs and ESCs were also active in SSCs (477, SME enhancers; Fig. 5a). A DNA motif search with HOMER revealed that many ESC signature transcription factors (for example, *Sox2* and *Pou5f1*) could bind at these common active enhancers, indicating that an ESC-like transcriptional programme might be partially active in SSCs (Fig. 5c and Supplementary Data 7). This notion was supported by comparison with published ChIP-seq data in ESCs^39^,^40^, demonstrating that the ME and SME enhancers we identified were highly occupied by many ESC expressed transcription factors, particularly those at the centre of the pluripotency circuitry (*Pou5f1*, *Sox2* and *Nanog*; Fig. 5d, Supplementary Fig. 9 and Supplementary Data 6). Based on the ESC data, these transcription factors had the potential to bind at enhancers near both active genes (Class I) and repressed genes (Class II) in MASCs, but could preferentially occupy the promoters of active genes (Class I) (Fig. 5c,d). Conversely, the promoters of repressed genes (Class II) were potential targets of ESC differentiation regulators that lose expression in MASCs (for example, *Egr1/2*, *Rfx1*) (Fig. 5c and Supplementary Data 7), as well as the PRC2 subunit *Suz12* that mediates K27me3 modification (Fig. 5d and Supplementary Fig. 9)^39^,^40^.

Unlike shared active enhancers in MASCs and ESCs (ME and SME enhancers), the 1,581 ESC-specific enhancers (E enhancers) were potential targets of many regulators of late embryogenesis (for example, *Usf1/2* and *Gata2/4*) but not the core pluripotency transcription factors (*Pou5f1*, *Sox2* and *Nanog*), suggesting that the ESC differentiation apparatus rather than the pluripotency circuitry could be responsible for the activation of such enhancers (Fig. 5c,d). We also found the motifs of several transcription factors (for example, *Prdm14* and *Foxh1*) or signalling regulators (for example, *Smad3*) enriched at MASC-specific enhancers (M enhancers), indicating their potential roles in SSC conversion (Fig. 5c).

In summary, MASCs underwent erasure of germline-specific enhancers and partial establishment of enhancers that resemble those of ESCs. This process would be prospectively enabled by binding of ESC signature transcription factors. The change in enhancer activity closely correlated with alterations in expression of nearby genes and could support SSC reprogramming along with changes in promoter chromatin state. In contrast to the regulation at enhancers, bivalently marked somatic gene promoters could be modulated by both PRC2 (*Suz12*) and target-specific transcription factors, with cooperative effects on active transcription or silencing of these genes, respectively, in a cell-type-specific manner.

### Promoter bivalency shared between SSCs and germ cells *in vivo*

Recently, global profiling of the germline epigenome revealed that the K4me3+K27me3 bivalent histone modification remains stable from the development of embryonic progenitors through to postnatal germ cells^24^,^41^,^42^. In addition to germ cell-specific genes, many developmental regulators functioning in somatic lineages are poised with both K4me3 and K27me3 histone modifications at promoters in PGCs^24^,^42^, adult germline stem cells^35^, pachytene spermatocytes^24^,^27^, round spermatids^24^,^26^,^27^ and mature spermatozoa^26^. This epigenetic feature is therefore suggested to be essential for germ cell identity and function^43^. To understand whether long-term *in vitro*-cultured SSCs preserve similar poised chromatin as germ cells *in vivo*, we compared our K4me3 and K27me3 ChIP-seq results from cultured SSCs with results of other published studies.

**Figure 6:**
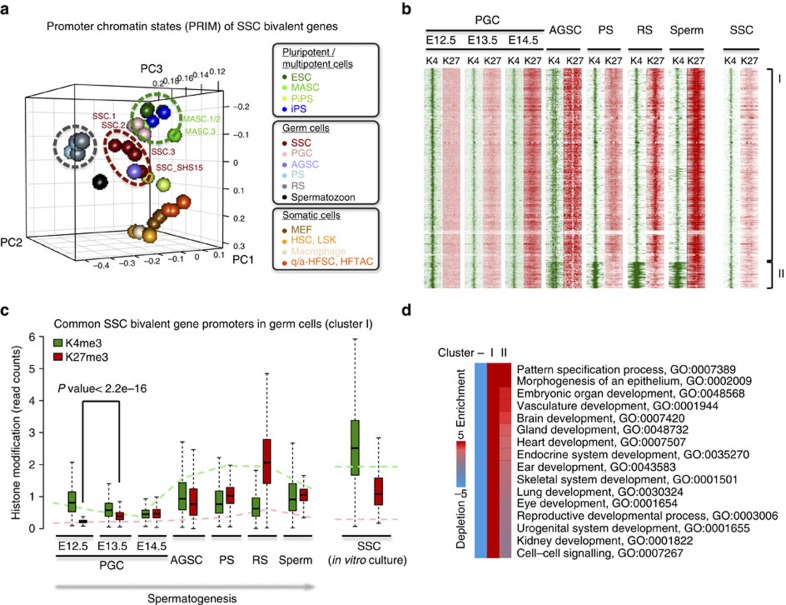
SSCs maintain consistent promoter bivalency with germ cells *in vivo*. (**a**) Three-dimensional (3D) PCA plot based on PRIMs of all promoters with K4me3+K27me3 bivalent histone modifications in SSCs. Different cell types are distinguished by colours as in Fig. 1. (**b**) *k*-means clustering of SSC bivalent genes by similarity of K4me3 and K27me3 profiles at promoters. Green, K4me3; red, K27me3. Cluster I, 2,644 genes; cluster II, 372 genes. (**c**) Histone modification profiling at promoters of all cluster I genes as grouped in **b**. Germ cells include those isolated from testis by fluorescent-activated cell sorting (left) and SSCs cultured *in vitro* (right). *y* axis, average read count within promoter region. Green box, K4me3 modification; red box, K27me3 modification. The bottom and top of the boxes indicate the 25th and 75th percentiles, the central bars indicate medians and whiskers indicate non-outlier extremes. *P* values were calculated using Wilcoxon tests. Dashed green line, average read count of K4me3 modification at all promoters in each cell type; dashed red line, average read count of K27me3 modification at all promoters in each cell type. (**d**) GO enrichment using iPAGE. Genes are grouped by promoter K4me3 and K27me3 profiles as in **b**.

We focused on 3,016 SSC bivalent genes, which were selected by peak detection as genes with significant K4me3 and K27me3 marks at promoters in at least two SSC cell lines (Supplementary Data 4). PCA analysis using PRIM values for these promoters showed that cultured SSCs not only shared similar K4me3- and K27me3-defined chromatin states with cultured MASCs and ESCs but also with PGCs directly isolated from embryonic gonads (E12.5–E14.5)^24^ (Fig. 6a and Supplementary Fig. 10). We next investigated histone modification changes at each promoter within the germline lineage. Using *k*-means clustering function in seqMINER^44^, SSC bivalent genes were grouped according to similarity of promoter K4me3 and K27me3 profiles in PGCs (E12.5, E13.5 and E14.5)^24^, adult germline stem cells^35^, pachytene spermatocytes^24^,^27^, round spermatids^24^,^26^,^27^ and mature spermatozoa^26^ (Fig. 6b and Supplementary Data 4). Notably, we found that nearly 90% of SSC bivalent genes (2,644, cluster I) maintained relatively high K4me3 and K27me3 modifications from PGCs to spermatozoa (Fig. 6b,c). The increase in K27me3 modification initiated as early as on E13.5 in PGCs, the time point of sex determination in the embryonic gonad, accelerated in postnatal testes and persisted at significant levels in spermatozoa (Fig. 6c). Furthermore, this class of genes was significantly enriched for developmental regulators that direct the differentiation of both germ and somatic cells, including *Sox2*, *Cdx2* and *Gata6*, among others (Fig. 6d and Supplementary Fig. 11). The K4me3 and K27me3 patterns at these developmental genes remained stable in different types of germ cells, but some of them resolved to K4me3 or K27me3 monovalent chromatin in somatic cells (MEFs; Supplementary Fig. 11). Importantly, these germline-poised developmental genes shared similar K4me3 and K27me3 modification patterns with cultured SSCs, providing support for the concept that germ cell identity is maintained in long-term *in vitro*-cultured SSC cell lines.

## Discussion

**Figure 7:**
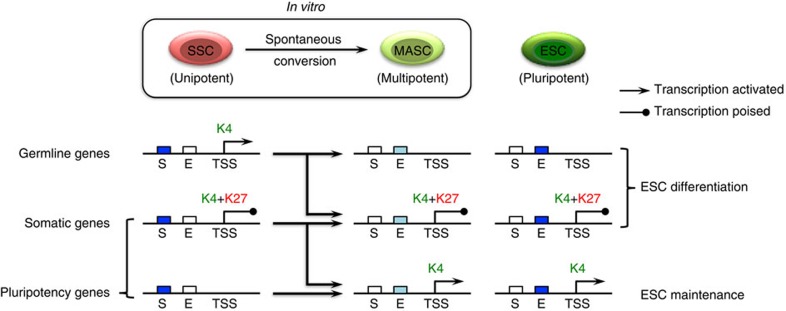
Model of epigenomic changes during SSC conversion to MASC. SSC reprogramming involves changes in the epigenome at both cell-type-specific promoters and enhancers. Together with gene reactivation and silencing in reprogrammed MASCs, K4me3 modification (K4) is enriched at the promoters of pluripotency-associated genes but depleted from promoters of germline-specific genes. Conversely, K27me3 modification (K27) is erased from the promoters of many genes that are expressed in ESCs. However, K27me3 modification ‘poises' both germline- and somatic-specific gene promoters together with K4me3 modification. At enhancer regions, MASCs are devoid of most germ cell signature enhancer activity (S) marked by K27ac modification but exhibit partially active ESC-specific enhancers (E). In summary, MASCs acquire an ESC-like epigenome at promoters and a subset of ESC-specific enhancers. Dark blue box, completely active enhancer; light blue box, partially active enhancer; white box, silent enhancer; TSS, transcription start site.

Here we provide comprehensive genome-wide maps of transcription-associated histone modifications in cultured mouse SSCs and their spontaneously converted counterparts (MASCs). In-depth analysis of the SSC epigenome, coupled with transcriptional profiling by RNA-seq and motif analysis, suggested that while the chromatin of somatic gene promoters exhibits stable bivalency, promoters and enhancers of signature genes for ESCs and germ cells undergo significant chromatin state changes after SSC conversion into a multipotent state (Fig. 7). This epigenomic alteration could play a critical role in SSC reprogramming. There are several important points that emerge from our study.

First, transcriptome analysis revealed that SSC reprogramming involves not only activation of somatic cell- and ESC-expressed genes but also repression of germ cell differentiation regulators. During early embryogenesis, many of these activated genes are expressed in the inner cell mass, the embryonic origin of ESCs, and are considered to be hallmarks of stem cell pluripotency. This suggests that SSCs reach a multipotent developmental state through both recovery of broad embryonic developmental programmes and erasure of adult germ cell characteristics.

Second, SSCs preserve ESC-like epigenetic features at both promoter and enhancer regions. In contrast to the differentiated cell types that we analysed, promoters of regulatory genes involved in embryonic differentiation maintained K4me3+K27me3 bivalent modifications in SSCs as in ESCs. Moreover, many ESC signature genes were also poised with bivalent modifications in SSCs. Furthermore, our motif study of active enhancers shared among SSCs, MASCs and ESCs suggests that the core pluripotency circuitry is partially active in SSCs. This unique epigenetic milieu could facilitate a transcriptional switch in the presence of internal or external stimuli and endow SSCs with unusual developmental flexibility with respect to lineage development beyond the germline.

Third, after SSC reprogramming, most pluripotency-associated gene promoters switch to active chromatin states through erasure of K27me3 modification (MASC^Active^) or acquisition of the K4me3 modification (MASC^Modified^). Conversely, promoters of germ cell differentiation genes are largely repressed by K27me3 modification (MASC^Repressive^) or silenced through loss of K4me3 modification (MASC^Unmodified^). Such a pattern suggests that H3K4- and H3K27-specific methyltransferases and demethylases may play active roles in SSC reprogramming, by facilitating chromatin state changes at selected promoters. Further study of enzymes that mediate H3K4 and H3K27 covalent modifications, particularly those specifically expressed in SSCs, may be useful to improve reprogramming efficiency.

Fourth, somatic genes generally retain bivalent promoter modifications in MASCs, despite changes in expression after SSC reprogramming (MASC^Stable I^ and MASC^Stable II^). Transcription of these poised genes is potentially regulated by both PRC2 (*Suz12*)-mediated K27me3 and by transcription factors at both promoters and enhancers. Many such transcription factors undergo significant increases in expression (class I) or decreases in expression (class II), as well as chromatin state changes during reprogramming, for example, the core pluripotency regulators (*Pou5f1*, *Sox2* and *Nanog*) and several ESCs differentiation factors. Our results suggest that epigenetic switches could associate with the expression changes of key transcription factors and initiate SSC reprogramming, while somatic genes represent downstream targets of this hierarchical network^45^. However, we cannot exclude the possibility that epigenetically poised somatic genes respond directly to the signalling associated with reprogramming, and these somatic genes change their expression levels before the chromatin state changes at pluripotency- and spermatogenesis-associated genes. Stepwise observations throughout the entire SSC reprogramming process, together with in-depth analysis of the transcriptome and associated epigenome, will be necessary to fully understand the mechanism of this unique reprogramming event.

Fifth, MASCs are depleted of germ cell-specific epigenetic signatures at most *cis*-regulatory regions, instead acquiring ESC-like promoter chromatin modifications and partially activating pluripotent ESC-specific enhancers. Because MASCs are capable of differentiating into all germ layers during teratoma formation, this finding suggests that promoter histone modifications, together with enhancers shared between MASCs and ESCs, are sufficient to support ESC-like tri-lineage differentiation, while those enhancers that remain silent in MASCs are not essential to the core pluripotency circuitry but could be induced during long-term culture. Thus, it will be important to investigate whether the establishment of fully embryonic-like enhancer activity improves developmental potency of cells to multiple lineages. As ESC-like cells derived from SSCs are known to be inefficient in both chimera contribution and germline transmission, and completely lack tetraploid complementation ability^1^,^2^, one possible solution is to improve the K27ac-defined global chromatin state in culture by histone deacetylases inhibitors (for example, valproic acid (VPA))^46^.

Finally, our study in different types of somatic and germ cells shows that ESC-like epigenetic characteristics are not only preserved in long-term *in vitro*-cultured SSCs but also in differentiating germ cells and mature spermatozoa *in vivo*. This result reinforces the recent reports that a set of developmental gene promoters are poised with K4me3+K27me3 bivalent modifications in mammalian germline, from PGCs, the embryonic progenitors of SSCs, to mature spermatozoa^24^,^41^,^42^. Our findings in SSCs further confirm the developmental plasticity of the adult germ cells. Furthermore, our data show that long-term *in vitro*-cultured SSCs preserve chromatin modifications at selected promoters as do germline progenitor cells *in vivo*. The remarkable chromatin consistency at developmental gene promoters suggests that germline epigenome could ensure stable transfer of epigenetic ‘memory' to the next generation, despite the global epigenomic changes in the embryonic gonad and during meiosis. Further study of these genes with germline-stable and -specific epigenetic marks will shed light on our understanding of the initiation of embryogenesis.

Our results offer insight into transcription factor-independent epigenetic regulation during mammalian cell reprogramming from a unipotent to a multipotent state and suggest several strategies to increase SSC reprogramming efficiency. Development of novel, transcription factor-free, enforced reprogramming strategies will greatly benefit stem cell application in the clinic, and will also shed light on the origin of totipotency during development.

## Methods

### Mice

SSCs were prepared from mice over 3 months old from the following strains: *Pou5f1-GFP JAXR* (stock number 004654) (OG)^47^; *C57Bl6* (B6), *C57Bl6/129S* mix (129mix)^2^; and *Gt(ROSA26)Sor-lacZ* (Rosa). All mouse experiments were performed in accordance with institutional and national guidelines and regulations including the Weill Cornell Medical College Institutional Animal Care and Use Committee.

### Cell culture

Mouse SSCs were cultured with standard protocol^2^. In brief, seminiferous tubules were collected from detunicated testes and minced on ice. The tissue was enzymatically dissociated with agitation for 30 min at 37 °C in a buffer containing 0.017% trypsin (Cellgro), 17 mM EDTA (Cellgro), 0.03% collagenase (Sigma-Aldrich) and DNase I (100 μg ml^−1^; Sigma-Aldrich). The cell suspension was then collected and plated in gelatin-coated plate in SSC medium containing StemPro-34 (Invitrogen) and supplements as follows: D(+) glucose, 6 mg ml^−1^; BSA, 0.50%; insulin, 25 μg ml^−1^ (Sigma-Aldrich); MEM nonessential amino acids, 1 × (Gibco); MEM vitamin solution, 1 × (Gibco); penicillin (100 U ml^−1^)/streptomycin (100 μg ml^−1^)/amphotericin (0.2 μg ml^−1^) (Media-tech); fetal bovine serum, 1%; L-glutamine, 2 mM (Media-tech); bovine holo-transferrin, 100 μg ml^−1^ (Sigma-Aldrich); β-oestradiol, 30 ng ml^−1^ (Calbiochem); progesterone, 60 ng ml^−1^ (Calbiochem); putrescine, 60 μM (Research Organics); sodium selenite, 30 nM (Sigma-Aldrich); pyruvic acid, 30 μg ml^−1^ (Sigma-Aldrich); D(L)-lactic acid, 1 μg ml^−1^ (Baker); β-mercaptoethanol, 50 μM (Gibco); ascorbic acid, 100 μM (EMD); D-biotin, 10 μg ml^−1^ (Calbiochem); human glial cell-derived neurotrophic factor (GDNF), 10 ng ml^−1^ (R&D Systems); human basic fibroblast growth factor, 10 ng ml^−1^ (Cell Signalling); and mouse epidermal growth factor, 20 ng ml^−1^ (R&D Systems). SSC colonies start to appear after about 1 week. The primary SSCs were then transferred to MEF (Millipore #PMEF-CF)-coated plate and expanded. The SSC identities of cultured cells were evaluated by several cell surface antigens (Supplementary Fig. 12). After at least 10 passages, SSCs were collected by gentle trituration of colonies attached to feeder cells or floating in the medium. To remove residual feeder cells in the collection, SSCs were plated in gelatin-coated plates in fresh SSC medium for 2 h. All the floating cells were then gently collected, washed once in PBS and subjected to ChIP experiments or frozen for RNA isolation.

MASCs were derived from SSC conversion after long-term *in vitro* expansion with standard SSC culture procedures. iPS cell lines were generated from E13.5 *tetO-4F2A* MEFs as previously described^23^. Briefly, 5 × 10^5^ MEF cells were seeded in each well of a six-well plate and maintained in standard ESC culture medium with 2 μg ml^−1^ doxycycline for over 20 days for reprogramming. To establish MASC or iPS cell lines from corresponding primary reprogrammed cell culture, colonies with typical undifferentiatied ESC morphology were identified by phase microscopy and mechanically separated from the plate using Pasteur pipettes. MASCs, ESCs and iPS cells were maintained using standard ESC culture procedures. In brief, cells were cultured with MEFs in standard ESC medium containing KnockOut DMEM (Gibco) and supplements as follows: KnockOut Serum Replacement, 10% (Gibco); penicillin (100 U ml^−1^)/streptomycin (100 μg ml^−1^)/amphotericin (0.2 μg ml^−1^) (Media-tech); L-glutamine, 2 mM (Media-tech); MEM nonessential amino acids, 1 × (Gibco); β-mercaptoethanol, 50 μM (Gibco); and ESGRO leukaemia inhibitory factor, 1,000 U ml^−1^ (Millipore). All the cells applied to further experiments were serially passaged and collected within 20 passages.

To expand mouse Lin^−^ HSCs, whole bone marrow cells from C57BL/6J mice were isolated and Lin^−^ cells were enriched by mouse Lineage Cell Depletion Kit (Miltenyi Biotec). Purified cells were plated with E4ORF1-HUVECs in X-Vivo serum-free media (Lonza) supplemented with 10 ng ml^−1^ of soluble Kit Ligand (sKitL) (Biosource)^48^. Total expanded cells were collected, enriched for Lin^−^ cells and plated with new E4ORF1-HUVECs feeders every 7 days. Total haematopoietic cell expansion was enriched for Lin^−^ cells by mouse Lineage Cell Depletion Kit before collection.

### ChIP and antibodies

ChIP was performed as previously described^49^. Briefly, 1 × 10^7^ cells per experiment were crosslinked for 15 min in 1% paraformaldehyde, washed and lysed. Chromatin was sheared using a Bioruptor to create fragments of ∼150 bp (base pairs), incubated with about 2–5 μg antibody bound to 75 μl Dynabeads M-280 (Invitrogen) and rotated overnight at 4 °C, then washed and eluted. The eluted chromatin was reverse-crosslinked and column-purified. ChIP was performed using the following antibodies: H3K4me3 (Abcam ab8580); H3K27me3 (Abcam ab6002); and H3K27ac (Abcam ab4729).

### ChIP-seq library construction and sequencing

ChIP samples were prepared for sequencing using Illumina TruSeq DNA Sample Preparation Kit according to the standard preparation protocol (http://www.illumina.com/). Sequencing service was performed on an Illumina Hiseq 2000 sequencer according to the standard Illumina protocol.

### RNA-seq library construction and sequencing

RNA was isolated using QIAGEN RNeasy kit. RNA samples were prepared for sequencing using Illumina TruSeq RNA Sample Preparation Kit and were sequenced on an Illumina HiSeq 2000.

### ChIP-seq data processing and analysis

ChIP-seq reads were aligned to the reference mouse genome (mm9, NCBI Build 37) using the BWA programme (version 0.5.9)^50^, and PCR duplicates were removed by Picard (version 1.69; http://picard.sourceforge.net/). Unique reads that mapped to a single best-matching location with no more than 4% of the read length of mismatches were kept and used to study genome-wide enrichment of specific histone modification. Sequence data were visualized with IGV by normalizing to 1 million reads^51^. Referenced data sets from publications: GEO repository: GSE12241, GSE47950, GSE42155, GSE11074, GSE69946, GSE55060, GSE49621, GSE42629, GSE22075, GSE29278, GSE31239, GSE24165, GSE11431 and GSE19019; Sequence Read Archive (SRA) repository: SRA097278.

The software ChIPseeqer-2.0 was applied to the ChIP-seq data with sequencing data from input DNA as control to identify genomic enrichment (peak) of specific histone modifications^36^. Promoters were defined as ±2 kb from transcription start site. The promoter chromatin state was determined by overlapping with significant K4me3 and/or K27me3 peaks (*t*=5, with *t* as the significance negative log *P* value (ratio) threshold for peaks) (FDR<0.05). Cell-type-specific bivalent genes were identified as promoters carrying both K4me3 and K27me3 peaks in at least two biological repeats of certain cell type. PRIM K4me3 and K27me3 (PRIMs) was calculated as 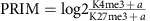, with K4me3 and K27me3 as average read counts from K4me3 and K27me3 sequences at the same promoter region and constant ‘*a*' equals to 0.001. Only promoters with detectable K4me3 or K27me3 peaks were evaluated by PRIMs.

An enhancer region was defined as any genomic locus with H3K27ac enrichment but without K4me3 enrichment (K27ac+K4me3−). To identify enhancer loci, K4me3 peaks were extended 1 kb each way. All H3K27ac peaks not overlapping with extended K4me3 peaks, known gene body, transcription start and end site were selected. Selected H3K27ac peaks within a 500-bp interval were merged together as enhancers. Enhancer regions identified from SSC, MASC and ESC^21^ were merged together. Each enhancer was annotated with all transcripts within a distance of 100 kb from their transcription start or end sites. Any enhancer overlap with K27ac peaks in a given cell type was considered to be active in that cell type.

### RNA-seq data processing and analysis

Reads from RNA-seq were aligned to mouse genome version mm9 using TopHat^52^, and fragments per kilobase of transcript per million fragments mapped (FPKM) were identified using Cufflinks with upper-quartile and GC-normalization^53^. Duplicate reads and reads aligning to more than one location were excluded. Gene expression was reported as log2-transformed FPKM value for a total of 32,581 unique transcripts. With Limma R package and expression profiles generated in our labs, lists of the differentially expressed genes in the pair-wise comparisons among SSC, MASC and ESC were generated. A total of 1,651 genes with at least two folds (log2) of expression differences and statistical significances (*P* value <0.05) between any pair of the three cell types were selected. These gene expression values were used as input for hierarchical clustering (centred on gene, uncentered on cell type, centroid linkage) by Cluster 3.0 (ref. 54).

### Gene Ontology analysis

Gene Ontology (GO) analysis was done using iPAGE with mouse GO annotation database^55^. In all, 31,881 GO terms were examined. Only non-electronic annotations were used in iPAGE and only categories with <300 genes were analysed. IPAGE used randomized simulations to ensure FDR<5%.

### Motif analysis

Enrichment of known motifs within promoter and enhancer regions was analysed with HOMER with default parameters and a fragment size of 200 bp. All known motifs used in our study were defined by HOMER.

### Statistical analysis

Statistical analysis was performed in R (version 3.2.1) statistical framework^56^. R packages applied for analysis and graph include limma (3.20.8), rgl (0.95.1247), gplots (2.14.1), ggplot2 (1.0.0) and VennDiagram (1.6.7).

## Additional information

**Accession codes:** ChIP-seq and RNA-seq have been deposited in the Gene Expression Omnibus (GEO) database under accession code GSE78127 (RNA-seq) and GSE78129 (ChIP-seq).

**How to cite this article:** Liu, Y. *et al*. Epigenetic profiles signify cell fate plasticity in unipotent spermatogonial stem and progenitor cells. *Nat. Commun.* 7:11275 doi: 10.1038/ncomms11275 (2016).

## Supplementary Material

Supplementary Information Supplementary Figures 1-12

Supplementary Data 1 List of RNA-seq datasets showing number of biological replicates and data sources.

Supplementary Data 2 List of ChIP-seq datasets showing number of biological replicates and sources.

Supplementary Data 3 Promoters with histone modification peak.

Supplementary Data 4 Promoters with K4me3+K27me3 bivalent histone modification peaks in ESC, MASC, SSC, iPS, and MEF. (1, K4me3 only; 0, K4me3+K27me3; -1, K27me3 only; NA, No modification).

Supplementary Data 5 Genes selected by differential expression among SSC, MASC, and ESC.

Supplementary Data 6 Enhancers associated with differentially expressed genes among SSC, MASC, and ESC. (Number of transcription factor peaks within one enhancer is shown in the table).

Supplementary Data 7 Motif enrichment at selected promoters and cell type-specific enhancers.

## References

[b1] Kanatsu-Shinohara M. . Generation of pluripotent stem cells from neonatal mouse testis. Cell 119, 1001–1012 (2004). 15620358 10.1016/j.cell.2004.11.011

[b2] Seandel M. . Generation of functional multipotent adult stem cells from GPR125+ germline progenitors. Nature 449, 346–350 (2007). 17882221 10.1038/nature06129 PMC2935199

[b3] Takahashi K. & Yamanaka S. Induction of pluripotent stem cells from mouse embryonic and adult fibroblast cultures by defined factors. Cell 126, 663–676 (2006). 16904174 10.1016/j.cell.2006.07.024

[b4] Takahashi K. . Induction of pluripotent stem cells from adult human fibroblasts by defined factors. Cell 131, 861–872 (2007). 18035408 10.1016/j.cell.2007.11.019

[b5] Pesce M., Wang X., Wolgemuth D. J. & Scholer H. Differential expression of the Oct-4 transcription factor during mouse germ cell differentiation. Mech. Dev. 71, 89–98 (1998). 9507072 10.1016/s0925-4773(98)00002-1

[b6] Arnold K. . Sox2+ adult stem and progenitor cells are important for tissue regeneration and survival of mice. Stem Cell 9, 317–329 (2011). 10.1016/j.stem.2011.09.001 PMC3538360 21982232

[b7] Zheng K., Wu X., Kaestner K. H. & Wang P. J. The pluripotency factor LIN28 marks undifferentiated spermatogonia in mouse. BMC Dev. Biol. 9, 38 (2009). 19563657 10.1186/1471-213X-9-38 PMC2719617

[b8] Boyer L. A. . Core transcriptional regulatory circuitry in human embryonic stem cells. Cell 122, 947–956 (2005). 16153702 10.1016/j.cell.2005.08.020 PMC3006442

[b9] Loh Y.-H. . The Oct4 and Nanog transcription network regulates pluripotency in mouse embryonic stem cells. Nat. Genet. 38, 431–440 (2006). 16518401 10.1038/ng1760

[b10] Costoya J. A. . Essential role of Plzf in maintenance of spermatogonial stem cells. Nat. Genet. 36, 653–659 (2004). 15156143 10.1038/ng1367

[b11] Kuramochi-Miyagawa S. . DNA methylation of retrotransposon genes is regulated by Piwi family members MILI and MIWI2 in murine fetal testes. Genes Dev 22, 908–917 (2008). 18381894 10.1101/gad.1640708 PMC2279202

[b12] Kolterud A., Alenius M., Carlsson L. & Bohm S. The Lim homeobox gene Lhx2 is required for olfactory sensory neuron identity. Development 131, 5319–5326 (2004). 15456728 10.1242/dev.01416

[b13] Wang G. G., Allis C. D. & Chi P. Chromatin remodeling and cancer, part I: covalent histone modifications. Trends Mol. Med. 13, 363–372 (2007). 17822958 10.1016/j.molmed.2007.07.003

[b14] Wang G. G., Allis C. D. & Chi P. Chromatin remodeling and cancer, part II: ATP-dependent chromatin remodeling. Trends Mol. Med. 13, 373–380 (2007). 17822959 10.1016/j.molmed.2007.07.004 PMC4337864

[b15] Goldberg A., Allis C. & Bernstein E. Epigenetics: a landscape takes shape. Cell 128, 635–638 (2007). 17320500 10.1016/j.cell.2007.02.006

[b16] Surani M., Hayashi K. & Hajkova P. Genetic and epigenetic regulators of pluripotency. Cell 128, 747–762 (2007). 17320511 10.1016/j.cell.2007.02.010

[b17] Brownell J. E. . Tetrahymena histone acetyltransferase A: a homolog to yeast Gcn5p linking histone acetylation to gene activation. Cell 84, 843–851 (1996). 8601308 10.1016/s0092-8674(00)81063-6

[b18] Li B., Carey M. & Workman J. L. The role of chromatin during transcription. Cell 128, 707–719 (2007). 17320508 10.1016/j.cell.2007.01.015

[b19] Azuara V. . Chromatin signatures of pluripotent cell lines. Nat. Cell Biol. 8, 532–538 (2006). 16570078 10.1038/ncb1403

[b20] Bernstein B. . A Bivalent chromatin structure marks key developmental genes in embryonic stem cells. Cell 125, 315–326 (2006). 16630819 10.1016/j.cell.2006.02.041

[b21] Creyghton M. P. . Histone H3K27ac separates active from poised enhancers and predicts developmental state. Proc. Natl Acad. Sci. USA 107, 21931–21936 (2010). 21106759 10.1073/pnas.1016071107 PMC3003124

[b22] Zhou V. W., Goren A. & Bernstein B. E. Charting histone modifications and the functional organization of mammalian genomes. Nat. Rev. Genet. 12, 7–18 (2011). 21116306 10.1038/nrg2905

[b23] Carey B. W., Markoulaki S., Beard C., Hanna J. & Jaenisch R. Single-gene transgenic mouse strains for reprogramming adult somatic cells. Nat. Methods 7, 56–59 (2010). 20010831 10.1038/nmeth.1410 PMC3048025

[b24] Lesch B. J., Dokshin G. A., Young R. A., McCarrey J. R. & Page D. C. A set of genes critical to development is epigenetically poised in mouse germ cells from fetal stages through completion of meiosis. Proc. Natl Acad. Sci. USA 110, 16061–16066 (2013). 24043772 10.1073/pnas.1315204110 PMC3791702

[b25] Hasegawa K. . SCML2 establishes the male germline epigenome through regulation of histone H2A ubiquitination. Dev. Cell 32, 574–588 (2015). 25703348 10.1016/j.devcel.2015.01.014 PMC4391279

[b26] Erkek S. . Molecular determinants of nucleosome retention at CpG-rich sequences in mouse spermatozoa. Nat. Struct. Mol. Biol. 20, 868–875 (2013). 23770822 10.1038/nsmb.2599

[b27] Sin H.-S., Kartashov A. V., Hasegawa K., Barski A. & Namekawa S. H. Poised chromatin and bivalent domains facilitate the mitosis-to-meiosis transition in the male germline. BMC Biol. 13, 53 (2015). 26198001 10.1186/s12915-015-0159-8 PMC4508805

[b28] Shen Y., Ye Z., Kuan S., Lobanenkov V. V. & Ren B. A map of the cis-regulatory sequences in the mouse genome. Nature 488, 116–120 (2012). 22763441 10.1038/nature11243 PMC4041622

[b29] Mikkelsen T. S. . Genome-wide maps of chromatin state in pluripotent and lineage-committed cells. Nature 448, 553–560 (2007). 17603471 10.1038/nature06008 PMC2921165

[b30] Pico A. R., Levine S. S. & Boyer L. A. Dynamic and coordinated epigenetic regulation of developmental transitions in the cardiac lineage. Cell 151, 206–220 (2012). 22981692 10.1016/j.cell.2012.07.035 PMC3462286

[b31] Banaszynski L. A. . Hira-dependent histone H3.3 deposition facilitates PRC2 recruitment at developmental loci in ES cells. Cell 155, 107–120 (2013). 24074864 10.1016/j.cell.2013.08.061 PMC3838450

[b32] Mikkelsen T. S. . Dissecting direct reprogramming through integrative genomic analysis. Nature 454, 49–55 (2008). 18509334 10.1038/nature07056 PMC2754827

[b33] Adli M., Zhu J. & Bernstein B. E. Genome-wide chromatin maps derived from limited numbers of hematopoietic progenitors. Nat. Methods 7, 615–618 (2010). 20622861 10.1038/nmeth.1478 PMC2924612

[b34] Lien W.-H. . Genome-wide maps of histone modifications unwind in vivo chromatin states of the hair follicle lineage. Cell Stem Cell 9, 219–232 (2011). 21885018 10.1016/j.stem.2011.07.015 PMC3166618

[b35] Hammoud S. S. . Chromatin and transcription transitions of mammalian adult germline stem cells and spermatogenesis. Stem Cell 15, 239–253 (2014). 10.1016/j.stem.2014.04.006 24835570

[b36] Giannopoulou E. G. & Elemento O. An integrated ChIP-seq analysis platform with customizable workflows. BMC Bioinformatics 12, 277 (2011). 21736739 10.1186/1471-2105-12-277 PMC3145611

[b37] Matsui Y., Zsebo K. & Hogan B. L. Derivation of pluripotential embryonic stem cells from murine primordial germ cells in culture. Cell 70, 841–847 (1992). 1381289 10.1016/0092-8674(92)90317-6

[b38] Resnick J. L., Bixler L. S., Cheng L. & Donovan P. J. Long-term proliferation of mouse primordial germ cells in culture. Nature 359, 550–551 (1992). 1383830 10.1038/359550a0

[b39] Chen X. . Integration of external signalling pathways with the core transcriptional network in embryonic stem cells. Cell 133, 1106–1117 (2008). 18555785 10.1016/j.cell.2008.04.043

[b40] Heng J.-C. D. . The nuclear receptor Nr5a2 can replace Oct4 in the reprogramming of murine somatic cells to pluripotent cells. Cell Stem Cell 6, 167–174 (2010). 20096661 10.1016/j.stem.2009.12.009

[b41] Sachs M. . Bivalent chromatin marks developmental regulatory genes in the mouse embryonic germline *in vivo*. Cell Rep. 3, 1777–1784 (2013). 23727241 10.1016/j.celrep.2013.04.032 PMC3700580

[b42] Ng J.-H. . *In vivo* epigenomic profiling of germ cells reveals germ cell molecular signatures. Dev. Cell 24, 324–333 (2013). 23352811 10.1016/j.devcel.2012.12.011

[b43] Lesch B. J. & Page D. C. Poised chromatin in the mammalian germ line. Development 141, 3619–3626 (2014). 25249456 10.1242/dev.113027 PMC4197577

[b44] Ye T. . seqMINER: an integrated ChIP-seq data interpretation platform. Nucleic Acids Res. 39, e35 (2011). 21177645 10.1093/nar/gkq1287 PMC3064796

[b45] Buganim Y. . Single-cell expression analyses during cellular reprogramming reveal an early stochastic and a late hierarchic phase. Cell 150, 1209–1222 (2012). 22980981 10.1016/j.cell.2012.08.023 PMC3457656

[b46] Huangfu D. . Induction of pluripotent stem cells by defined factors is greatly improved by small-molecule compounds. Nat. Biotechnol. 26, 795–797 (2008). 18568017 10.1038/nbt1418 PMC6334647

[b47] Szabó P. E., Hübner K., Schöler H. & Mann J. R. Allele-specific expression of imprinted genes in mouse migratory primordial germ cells. Mech. Dev. 115, 157–160 (2002). 12049782 10.1016/s0925-4773(02)00087-4

[b48] Butler J. M. . Endothelial cells are essential for the self-renewal and repopulation of Notch-dependent hematopoietic stem cells. Cell Stem Cell 6, 251–264 (2010). 20207228 10.1016/j.stem.2010.02.001 PMC2866527

[b49] Goldberg A. D. . Distinct factors control histone variant H3.3 localization at specific genomic regions. Cell 140, 678–691 (2010). 20211137 10.1016/j.cell.2010.01.003 PMC2885838

[b50] Li H. & Durbin R. Fast and accurate short read alignment with Burrows-Wheeler transform. Bioinformatics 25, 1754–1760 (2009). 19451168 10.1093/bioinformatics/btp324 PMC2705234

[b51] Thorvaldsdóttir H., Robinson J. T. & Mesirov J. P. Integrative Genomics Viewer (IGV): high-performance genomics data visualization and exploration. Brief Bioinformatics 14, 178–192 (2013). 22517427 10.1093/bib/bbs017 PMC3603213

[b52] Trapnell C., Pachter L. & Salzberg S. L. TopHat: discovering splice junctions with RNA-Seq. Bioinformatics 25, 1105–1111 (2009). 19289445 10.1093/bioinformatics/btp120 PMC2672628

[b53] Trapnell C. . Transcript assembly and quantification by RNA-Seq reveals unannotated transcripts and isoform switching during cell differentiation. Nat. Biotechnol. 28, 516–520 (2010). 20436464 10.1038/nbt.1621 PMC3146043

[b54] de Hoon M. J. L., Imoto S., Nolan J. & Miyano S. Open source clustering software. Bioinformatics 20, 1453–1454 (2004). 14871861 10.1093/bioinformatics/bth078

[b55] Goodarzi H., Elemento O. & Tavazoie S. Revealing global regulatory perturbations across human cancers. Mol. Cell 36, 900–911 (2009). 20005852 10.1016/j.molcel.2009.11.016 PMC2900319

[b56] R Core Team. R: A language and environment for statistical computing. R Foundation for Statistical Computing, Vienna, Austria http://www.R-project.org/ (2015).

