# Supplementary material for: Epigenetic profiles signify cell fate plasticity in unipotent spermatogonial stem and progenitor cells
Source: Nat Commun. 2016 Apr 27;7:11275. doi: 10.1038/ncomms11275 (PMC4853422; doi:10.1038/ncomms11275)
Supplement: Supplementary Information — Supplementary Figures 1-12 [file ncomms11275-s1.pdf]

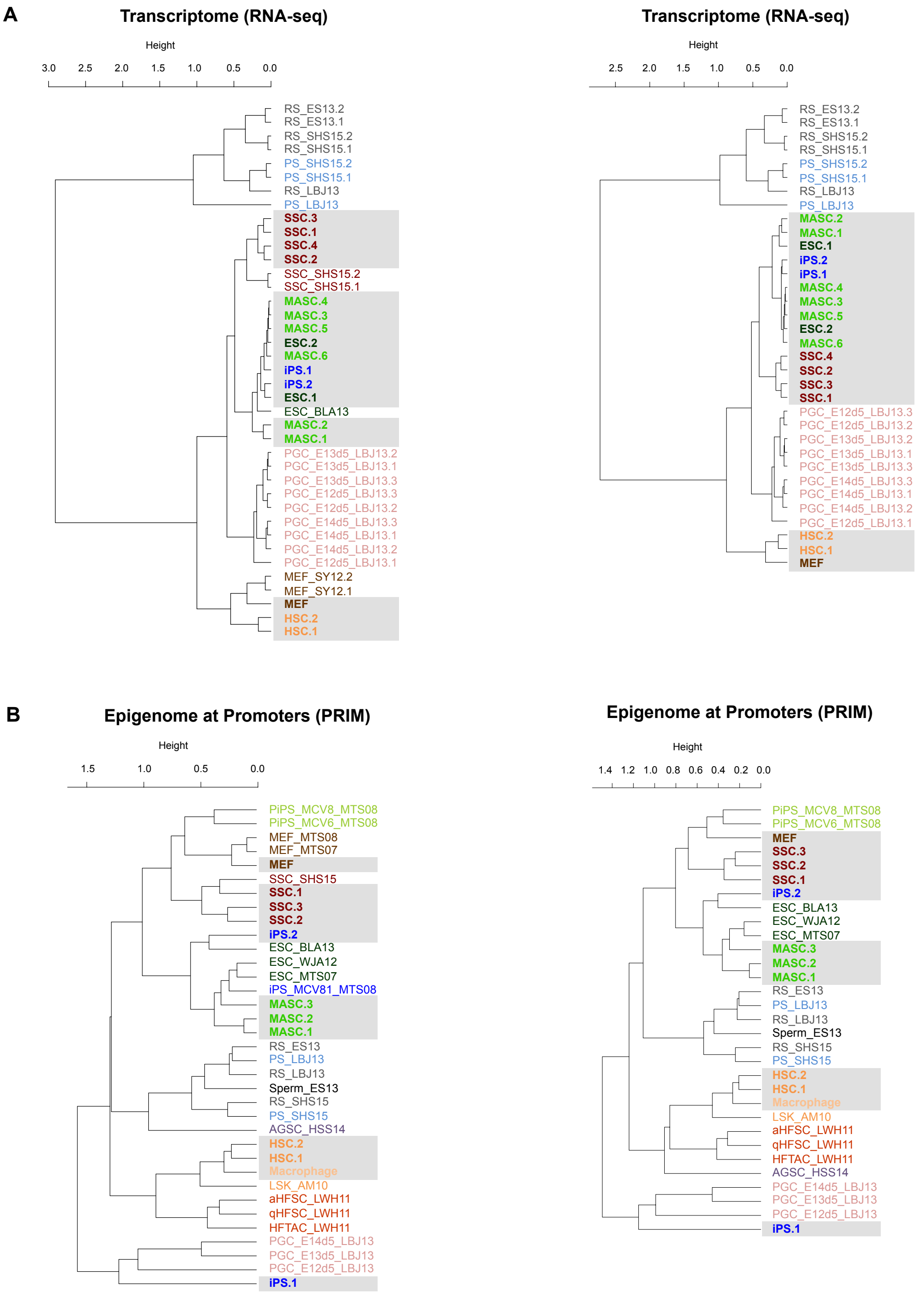

Supplementary Figure 1. Dendrogram based on (A) expression or (B) PRIMs of all protein-coding and noncoding genes. Different cell types are distinguished by colors as in Figure 1. Left, dendrograms include all cell lines. Right, dendrograms without published data on SSCs, iPSCs, and MEFs. In-house data are highlighted in gray.

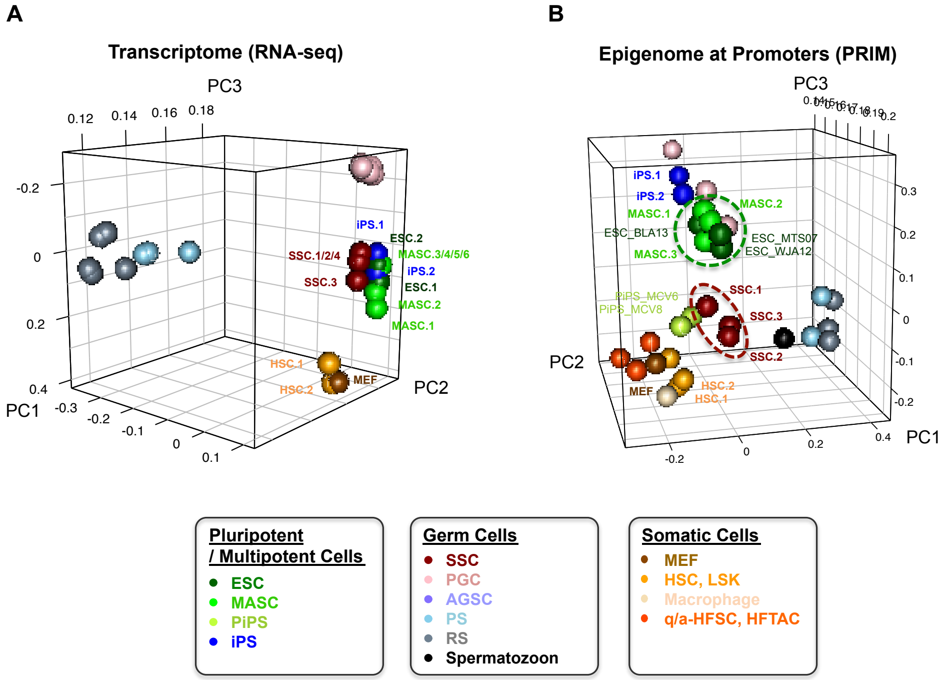

Supplementary Figure 2. 3D PCA plot based on (A) expression or (B) PRIMs of all protein-coding and noncoding genes. For SSCs, iPSCs, and MEFs, only in-house data were analyzed. Different cell types are distinguished by colors as in Figure 1.

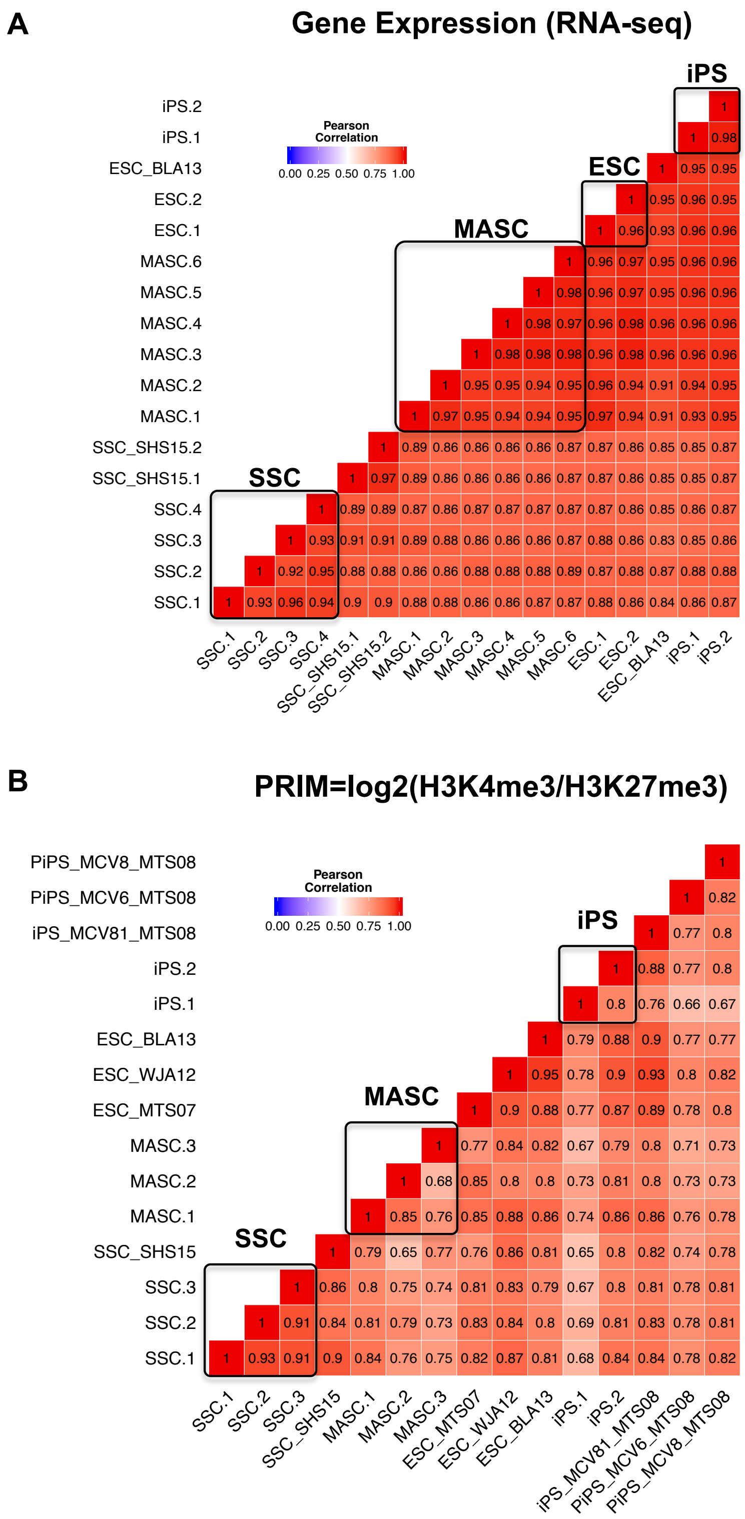

Supplementary Figure 3. Correlation coefficients between in-house and published cell lines. (A) Expression of all protein-coding and noncoding genes. (B) PRIMs of all protein-coding and noncoding gene promoters with K4me3 and/or K27me3 modification. Black boxes denote data produced in-house.

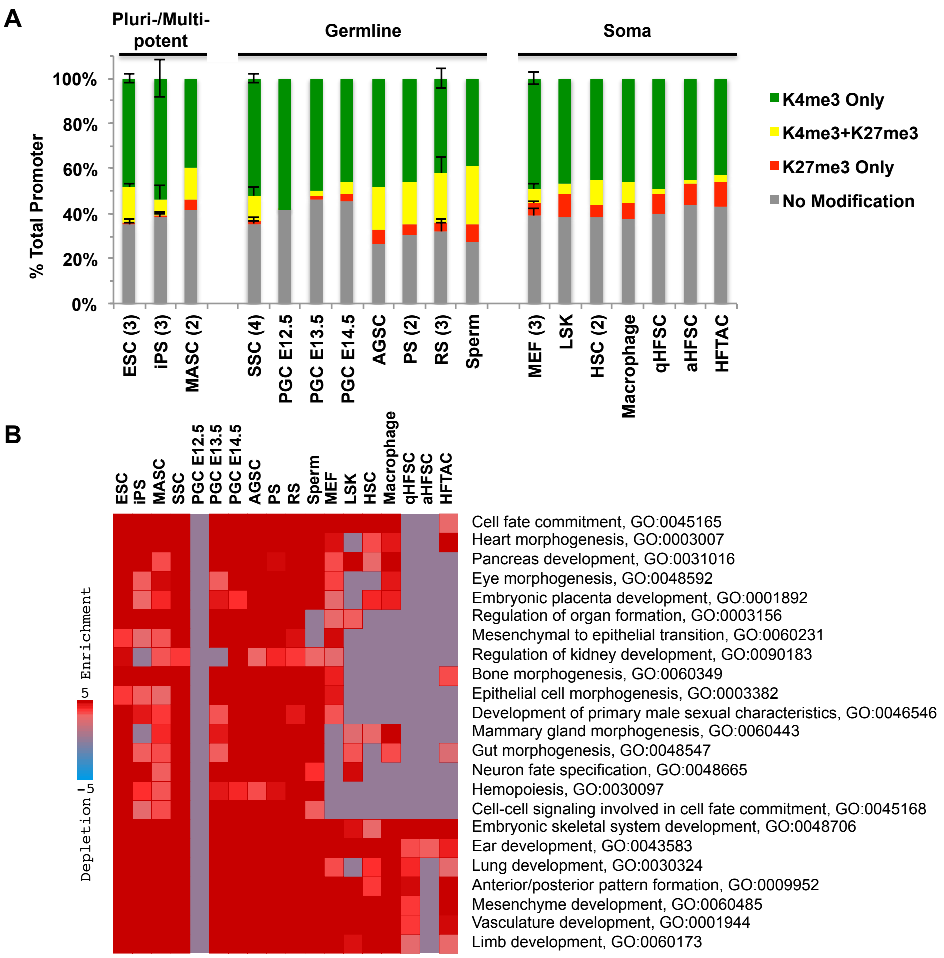

Supplementary Figure 4. Genes grouped by promoter histone modifications.

(A) Using peak detection results at promoters, all 32,581 protein-coding and noncoding genes were assigned to each of four different categories: K4me3 only (green), only marked by K4me3 modification; K27me3 only (red), only marked by K27me3 modification; K4me3+K27me3 (yellow), marked by both K4me3 and K27me3 modifications; and no modification (grey), no significant K4me3 or K27me3 modification detected. For each cell type with at least three biological repeats from different cell lines or resources (Supplementary Table S1), results are presented as mean values and standard deviations. Number in brackets, sample size.

(B) GO enrichment in bivalent genes identified from different cell types. Red, over representation. Blue, under representation.

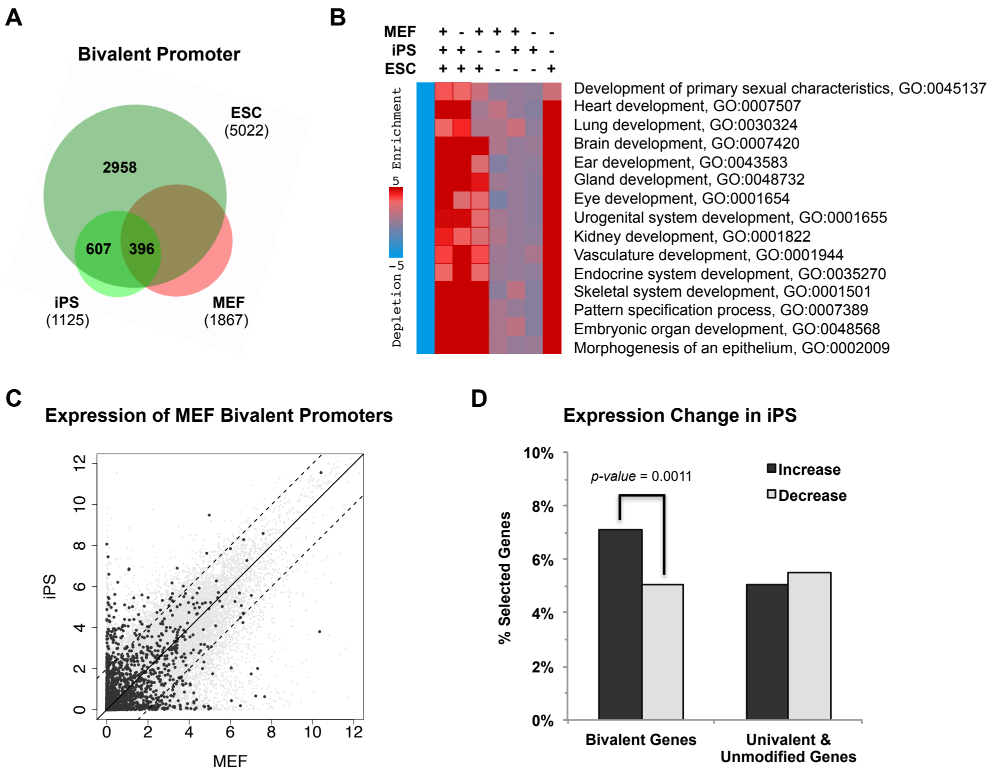

Supplementary Figure 5. ESC-like bivalent promoter modification is incompletely established in iPS.

(A) Overlap of bivalent genes identified by peak detection in ESCs, MEFs, and iPS cells.

(B) GO enrichment in bivalent genes shared or unique in ESCs, MEFs, and iPS cells. Presence and absence of promoter bivalency in certain cell types is denoted by "+" and "-", respectively. Red, over representation. Blue, under representation.

(C) Comparison of global gene expression profiles between MEFs and iPS cells. Black dots, MEF bivalent genes identified by peak detection. dashed line, cut-off of two-fold (log2) expression differences between MEFs and iPS cells.

(D) Percentage of genes with expression increases (black) or decreases (grey) in iPS cells. MEF bivalent genes, 1,867 genes with both K4me3 and K27me3 modifications at promoters. MEF univalent & unmodified genes, 30,714 genes modified with either K4me3, K27me3, or neither of the two modifications at promoters. *p-value* = 0.0011 by Fisher's exact test (one-sided).

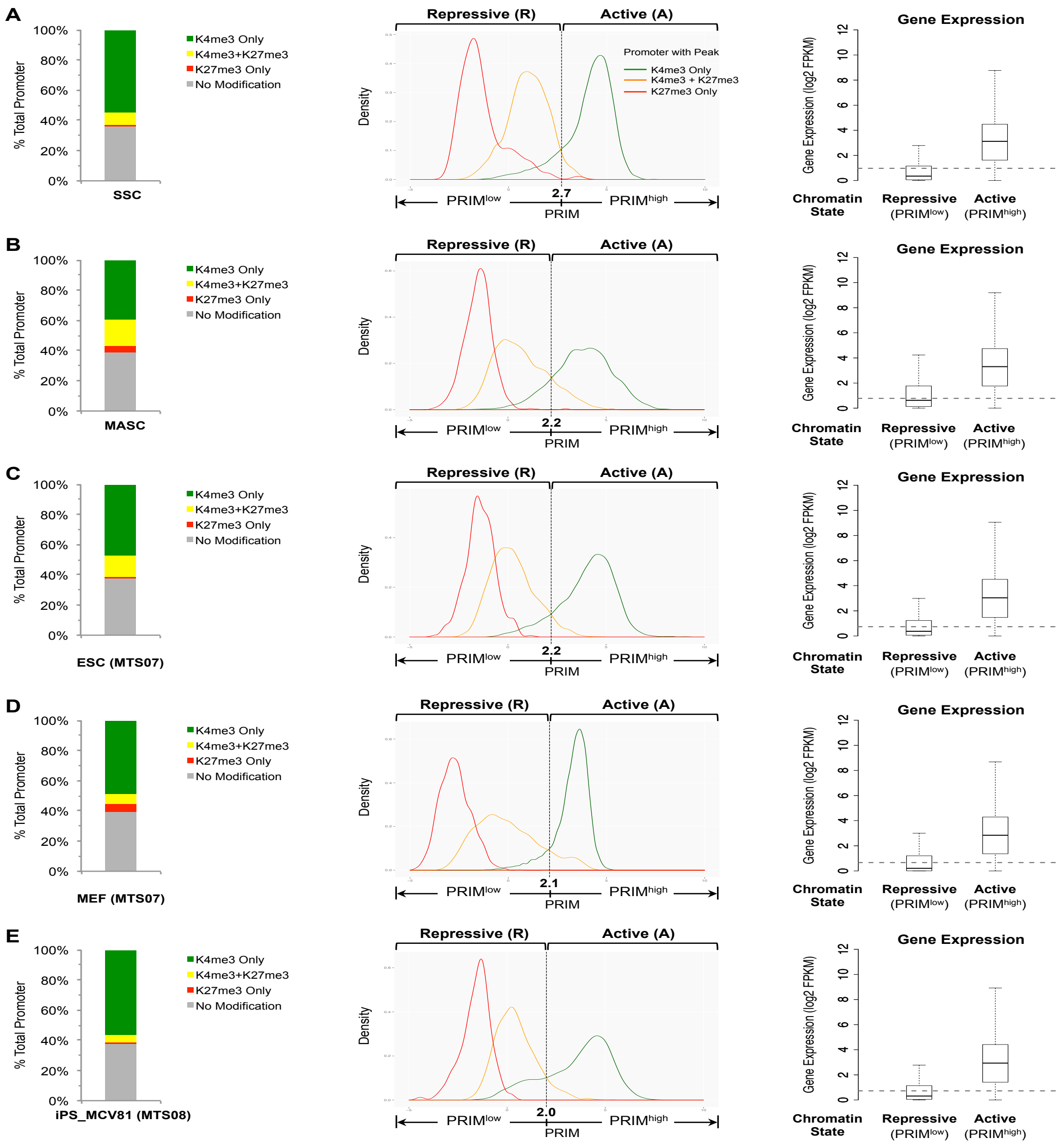

Supplementary Figure 6. Distinguishing active and repressive promoter chromatin states by PRIMs in (A) SSCs, (B) MASCs, (C) ESCs, (D) MEFs, and (E) iPS.
Based on peak detection results at promoters, all genes were assigned to each of four histone modification categories as in Figure S4A (left). Probability density distribution of PRIMs was made for each of the three categories, K4me3 only (green line), K4me3+K27me3 (yellow line), and K27me3 only (red line), and cut-off value between K4me3 and K4me3+K27me3 or K27me3 groups was selected by the crossover between K4me3 and K4me3+K27me3 plot (dashed line). X-axis, PRIMs. Y-axis, probability density. Cut-off value, SSC=2.7, MASC=2.2, ESC=2.2, MEF=2.1, iPS=2.0 (middle). Expression of genes in promoter chromatin states delineated by active (above PRIM cut-off) and repressive (at or below PRIM cut-off) marks is shown in boxplot (Right). The bottom and top of the boxes indicate the 25th and 75th percentiles, the central bars indicate medians and whiskers indicate non-outlier extremes. Dashed line, median expression value of all genes.

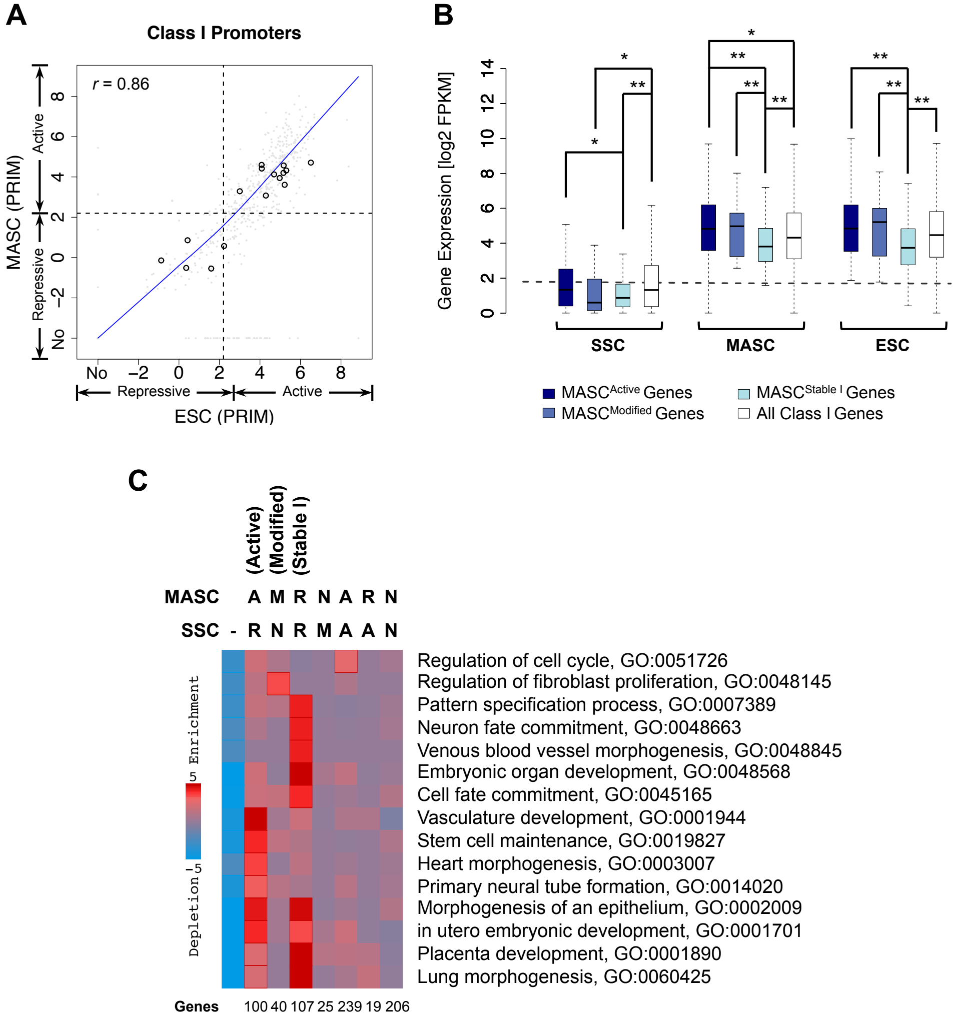

Supplementary Figure 7. Promoter chromatin states, transcriptional activities, and biological functions of genes activated during reprogramming of SSCs (Class I).

(A) Comparison of chromatin states (PRIMs) between ESCs and MASCs for all class I promoters (grey dots). X-axis, PRIMs from ESCs. Y-axis, PRIMs from MASCs. Black dots, class I example genes displayed in Figure 3A. Dashed black line, cut-off between active and repressive chromatin states. Dark grey line, optimal curve fits to all class I promoters. r, correlation coefficient with all class I promoter PRIMs.

(B) Transcriptional activities of selected gene subsets. Genes are grouped by promoter PRIMs as in Figure 4A. The bottom and top of the boxes indicate the 25th and 75th percentiles, the central bars indicate medians and whiskers indicate non-outlier extremes. *p-values* were calculated using Wilcoxon tests. **, *p-value* < 0.01. *, *p-value* < 0.05. Dashed line, median expression value of all genes.

(C) GO enrichment in all Class I genes. Genes are grouped by promoter PRIMs as in Figure 4A. A, Active chromatin state. R, Repressive chromatin state. M, Histone modification (K4me3 or K27me3) identifiable. N, Histone modification (K4me3 or K27me3) unidentifiable. Bottom, number of genes in each group.

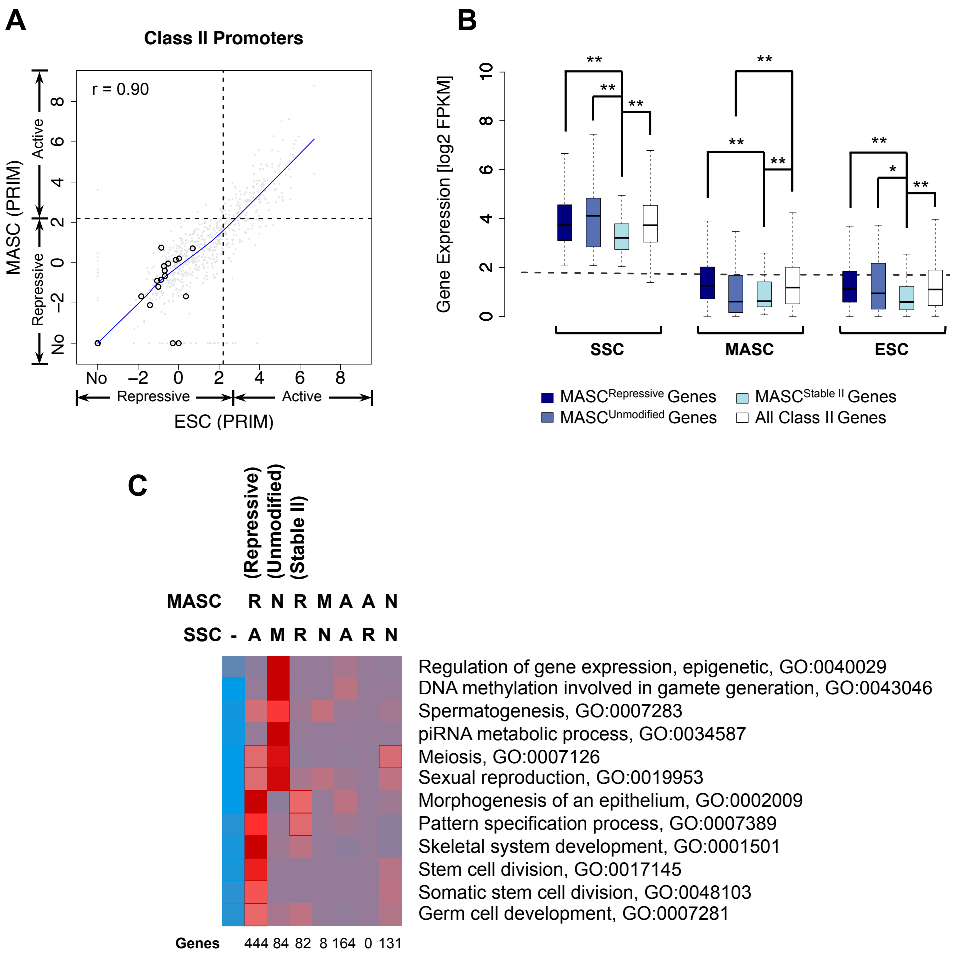

Supplementary Figure 8. Promoter chromatin states, transcriptional activities, and biological functions of gene repressed during reprogramming of SSCs (Class II).

(A) Comparison of chromatin states (PRIMs) between ESCs and MASCs for all class II promoters (grey dots). X-axis, PRIMs from ESCs. Y-axis, PRIMs from MASCs. Black dots, Class II example genes displayed in Figure 3A. Dashed black line, cut-off between active and repressive chromatin states. Dark grey line, optimal curve fits to all class II promoters. r, correlation coefficient with all class II promoter PRIMs.

(B) Transcription activities of selected gene subsets. Genes are grouped by promoter PRIMs as in Figure 4C. The bottom and top of the boxes indicate the 25th and 75th percentiles, the central bars indicate medians and whiskers indicate non-outlier extremes. *p-values* were calculated using Wilcoxon tests. **, *p-value* < 0.01. *, *p-value* < 0.05. Dashed line, median expression value of all genes.

(C) GO enrichment in all Class II genes. Genes are grouped by promoter PRIMs as in Figure 4C. A, active chromatin state. R, repressive chromatin state. M, histone modification (K4me3 or K27me3) is identifiable. N, histone modification (K4me3 or K27me3) is unidentifiable. Bottom, number of genes in each group.

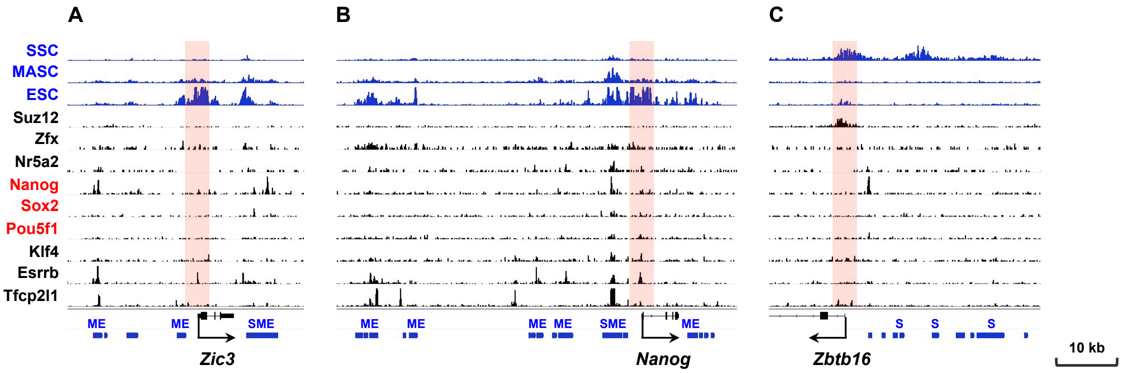

Supplementary Figure 9. K27ac modification in different cell types (blue) and transcription factor enrichment in ESCs (black) at selected genes. (A) *Zic3* (Class I, $MASC^{Active}$). (B) *Nanog* (Class I, $MASC^{Modified}$). (C) *Zbtb16* (Class II, $MASC^{Repressive}$). Core pluripotency regulators (Pou5f1, Sox2, Nanog) are highlighted in red. Pink region, Promoter. Blue bar at bottom, enhancer. ME, enhancers active in both MASCs and ESCs. SME, enhancers active in SSC, MASCs and ESCs. S, enhancers active only in SSCs. K27ac and transcription factor track range, 0 – 1.

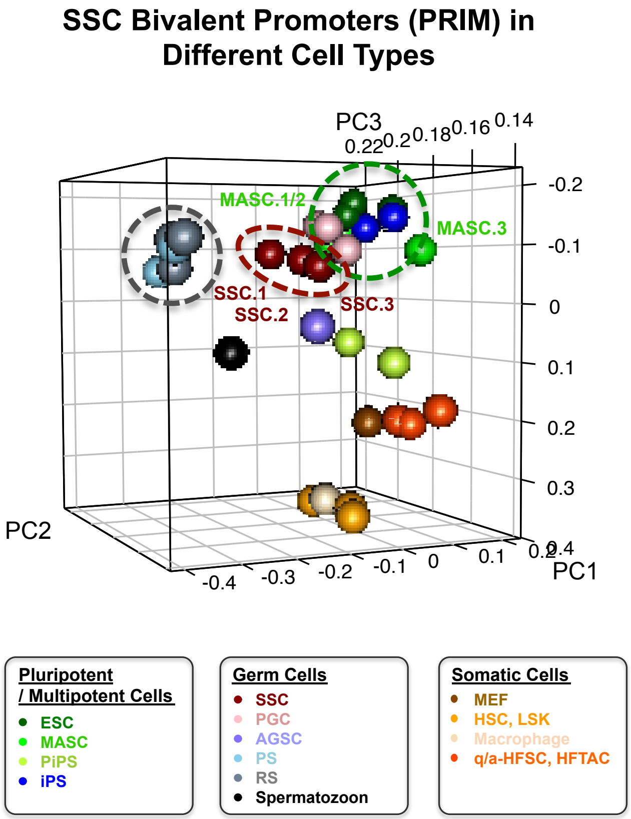

Supplementary Figure 10. 3D PCA plot based on PRIMs of all promoters with K4me3+K27me3 bivalent histone modifications in SSCs. For SSCs, iPSCs, and MEFs, only in-house data were analyzed. Different cell types are distinguished by colors as in Figure 1.

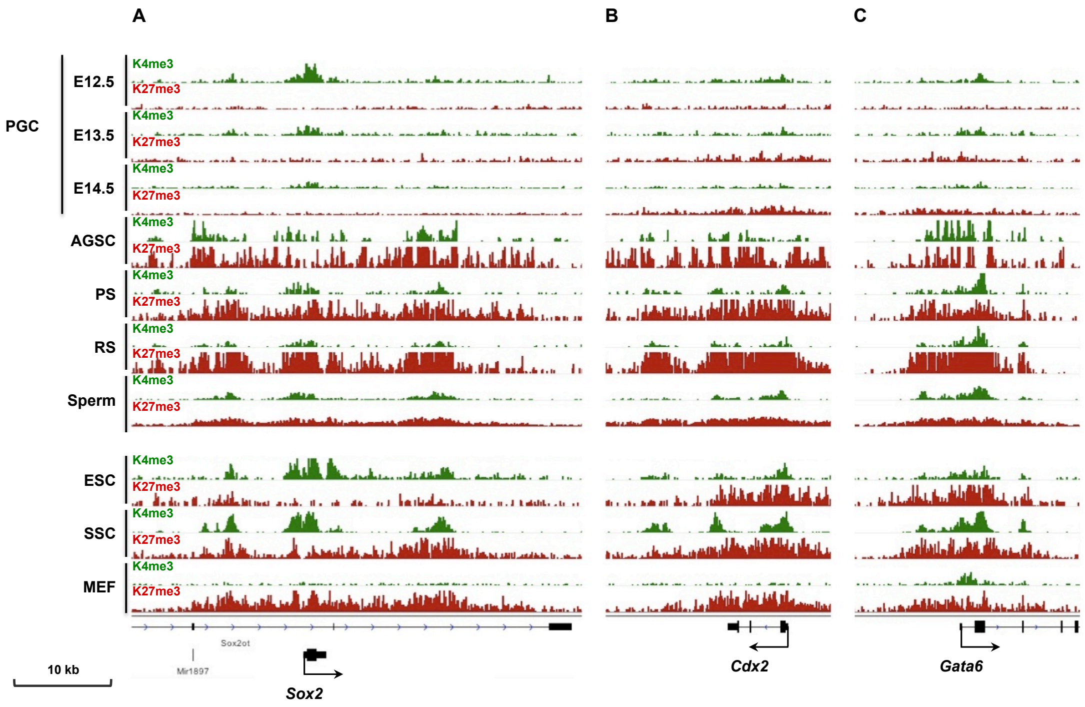

Supplementary Figure 11. Histone modifications at selected genes in different types of germ cells. Green, K4me3 modification. Red, K27me3 modification. K4me3 track range, 0 – 1. K27me3 track range, 0 – 0.5.

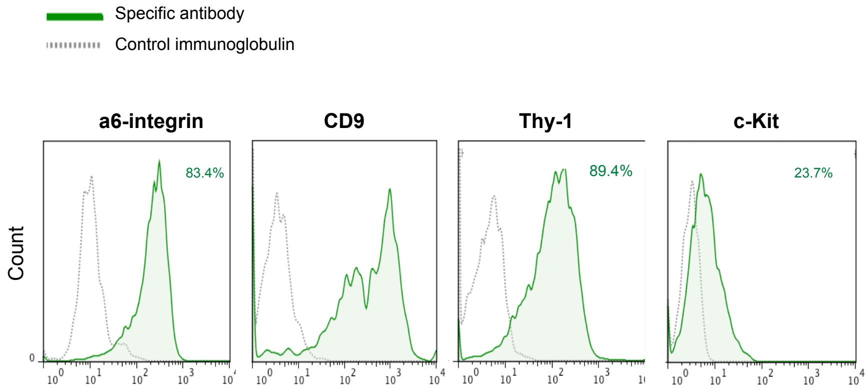

Supplementary Figure 12. Analysis of SSC cell surface marks by flow cytometry.
